# Dose-dependent responses to canonical Wnt transcriptional complexes in the regulation of mammalian nephron progenitors

**DOI:** 10.1242/dev.202279

**Published:** 2024-09-30

**Authors:** Helena Bugacov, Balint Der, Bohdana-Myroslava Briantseva, Qiuyu Guo, Sunghyun Kim, Nils O. Lindström, Andrew P. McMahon

**Affiliations:** ^1^ Department of Stem Cell Biology and Regenerative Medicine, Eli and Edythe Broad Center for Regenerative Medicine and Stem Cell Research, Keck School of Medicine of the University of Southern California, Los Angeles, CA 90089, USA; ^2^ Icahn School of Medicine at Mount Sinai, New York, NY 10029, USA; ^3^ Department of Urology, Faculty of Medicine, Semmelweis University, Budapest 1082, Hungary; ^4^ Institute of Translational Medicine, Faculty of Medicine, Semmelweis University, Budapest 1094, Hungary; ^5^ Discovery Biomarkers, Amgen Research, 1 Amgen Center Drive, Thousand Oaks, CA 91320, USA

**Keywords:** Nephron progenitor cells, Proliferation, Induction, Differentiation, Wnt/β-catenin signaling

## Abstract

*In vivo* and *in vitro* studies argue that concentration-dependent Wnt signaling regulates mammalian nephron progenitor cell (NPC) programs. Canonical Wnt signaling is regulated through the stabilization of β-catenin, a transcriptional co-activator when complexed with Lef/Tcf DNA-binding partners. Using the GSK3β inhibitor CHIR99021 (CHIR) to block GSK3β-dependent destruction of β-catenin, we examined dose-dependent responses to β-catenin in mouse NPCs, using mRNA transduction to modify gene expression. Low CHIR-dependent proliferation of NPCs was blocked on β-catenin removal, with evidence of NPCs arresting at the G2-M transition. While NPC identity was maintained following β-catenin removal, mRNA-seq identified low CHIR and β-catenin dependent genes. High CHIR activated nephrogenesis. Nephrogenic programming was dependent on Lef/Tcf factors and β-catenin transcriptional activity. Molecular and cellular features of early nephrogenesis were driven in the absence of CHIR by a mutated stabilized form of β-catenin. Chromatin association studies indicate low and high CHIR response genes are likely direct targets of canonical Wnt transcriptional complexes. Together, these studies provide evidence for concentration-dependent Wnt signaling in the regulation of NPCs and provide new insight into Wnt targets initiating mammalian nephrogenesis.

## Teaser

**Summary:** Rapid RNA-mediated genetic modification of nephron progenitor cells *in vitro* reveals dose-dependent responses to canonical Wnt pathway activity in the self-renewal and differentiation of mammalian nephron progenitor cells.

## INTRODUCTION

Wnt signaling is required for the self-renewal and differentiation of many key progenitor and stem cell types (Clevers et al., 2014). In canonical Wnt signaling, Ctnnb1/β-catenin transforms Wnt ligand binding to cell surface receptor complexes into a transcriptional output (Clevers et al., 2014). Stabilization of β-catenin, in response to canonical Wnt signaling through Frizzled receptor complexes, results in the translocation of β-catenin to the nucleus, where β-catenin interaction with Lef/Tcf DNA-binding partners at cis-regulatory modules results in the transcriptional activation of target gene transcription (Cadigan and Waterman, 2012; Mazzotta et al., 2016). In a non-transcriptional role, β-catenin is crucial for cadherin-mediated cell adhesion at adherens junctions. In this capacity, β-catenin is necessary for the formation and morphogenesis of epithelia, cell-cell recognition and the sorting of cell populations directed by distinct cell-surface cadherin complexes (Brembeck et al., 2006; Valenta et al., 2012).

The generation of a species-appropriate number of nephrons – approximately 14,000 in the mouse and one million in the human kidney – is crucially dependent on balancing the self-renewal and differentiation of nephron progenitor cells (NPCs) (McMahon, 2016). In these events, Wnt9b-directed canonical Wnt signaling through β-catenin is implicated in both the control of the NPC self-renewal state and the counter process of differentiation of NPCs (Brown et al., 2015; Carroll et al., 2005; Karner et al., 2011; Park et al., 2012, 2007; Ramalingam et al., 2018; Tanigawa et al., 2016). In this inductive process, Wnt9b signaling initiates a transcriptional program of active nephrogenesis and the associated cellular transition of mesenchymal NPCs into an epithelial renal vesicle, which is the precursor for each nephron (McMahon, 2016).

The development of an *in vitro* model for the culture of mouse NPCs enables NPC regulation to be studied in a controlled environment that mirrors properties of the normal nephrogenic niche (Brown et al., 2015; Li et al., 2019; Tanigawa et al., 2015, 2016). In nephron progenitor expansion medium (NPEM; Brown et al., 2015), Wnt signaling input is indirectly controlled by modulating β-catenin levels through varying levels of CHIR99021 (hereafter referred to as CHIR), a small molecule inhibitor of glycogen synthase kinase-β (GSK3β; Bennett et al., 2002). GSK3β-directed phosphorylation of β-catenin controls cytoplasmic levels of β-catenin through the activity of a destruction complex (Zhang et al., 2023). *In vivo* and *in vitro* analysis of NPCs has demonstrated β-catenin-associated enhancers linked to genes active within NPCs or activated at the onset of nephrogenesis (Guo et al., 2021; Karner et al., 2011; Park et al., 2012; Ramalingam et al., 2018). Genome-scale chromatin interaction studies, and subsequent validation of predicted Lef/Tcf/ β-catenin-dependent *cis*-regulatory modules, provide strong support for β-catenin in transcriptional activation of target genes in the induction of NPCs (Guo et al., 2021; Park et al., 2012). In contrast, the data are less clear for the action of β-catenin in the NPC program (Karner et al., 2011; Ramalingam et al., 2018; Guo et al., 2021).

Using an approach for rapid, RNA-mediated genetic modification within NPCs *in vitro*, we analyzed canonical Wnt pathway activity in the self-renewal and induction of primary mouse NPCs. Given the breadth of Wnt regulation of stem/progenitor programs (Lien and Fuchs, 2014; Loh et al., 2016; Rim et al., 2022), these findings may have significance beyond the mammalian kidney.

## RESULTS

### NPEM culture provides a rapid and consistent method for studying Wnt-supported NPC self-renewal and differentiation

**Fig. 1.**
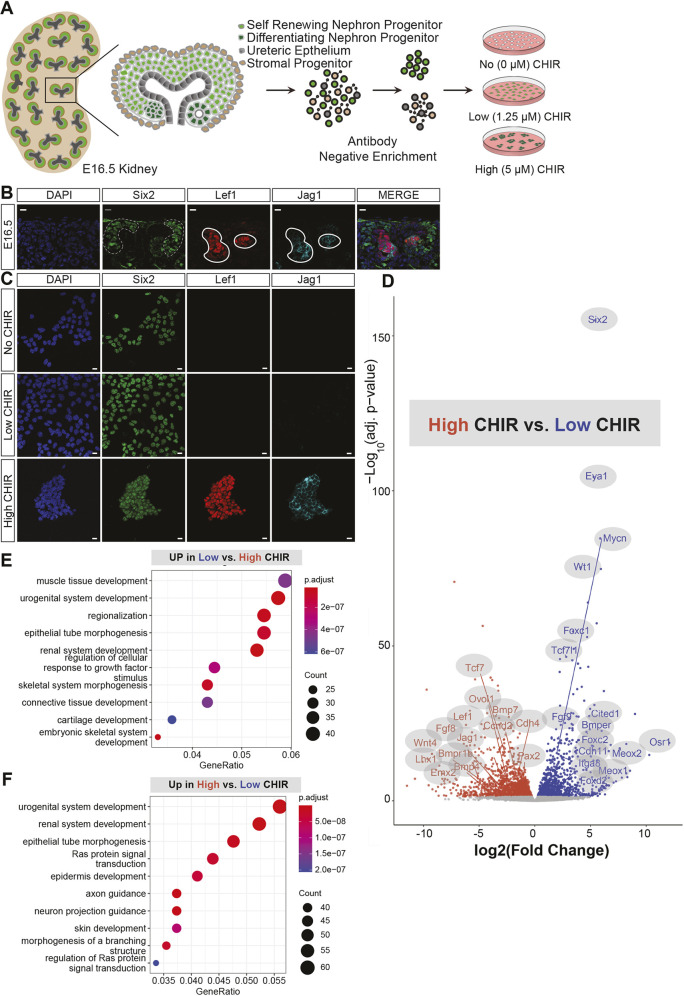
**Nephron progenitor expansion medium culture provides a rapid and consistent method for studying Wnt-supported nephron progenitor cell self-renewal and differentiation.** (A) Schematic representation of nephron progenitor cell (NPC) isolation and culture in nephron progenitor expansion medium (NPEM) supplemented with no (0 μM) CHIR, low (1.25 μM) CHIR and high (5 μM) CHIR. (B) Immunofluorescent staining for Six2, Lef1 and Jag1, and with DAPI in E16.5 wild-type kidney. Scale bars: 10 μm. Dotted lines outline a self-renewing area of the nephrogenic zone marked by high levels of Six2 protein; solid lines outline differentiating NPCs in the nephrogenic zone with high levels of Lef1 and Jag1. (C) Immunofluorescence staining for Six2, Lef1 and Jag1, and with DAPI on wild-type NPCs culture in no (0 μm), low (1.25 μm) and high (5 μm) CHIR). Scale bars: 10 μm. (D) Bulk RNA-seq. data of DEGs using Log2FC=absolute value cut-off 0.5 and *P*-adjusted value cut-off=0.05 comparing low versus high after 24 h of culture represented as a volcano plot. (E) Top 10 most significant GO analysis of DEGs that are upregulated in low CHIR compared with high CHIR. (F) GO analysis of DEGs that are upregulated in high CHIR compared with low CHIR.

To model the self-renewal and differentiation of NPCs *in vitro*, primary mouse NPCs were isolated from embryonic day 16.5 (E16.5) kidneys by enzymatic digestion and magnetic depletion (Fig. 1A), and cultured in NPEM: a defined medium formulated on signaling interactions in the nephrogenic niche (Brown et al., 2015). NPC expansion over multiple generations requires low levels of the GSK3β inhibitor CHIR (1.25 µM; hereafter low CHIR). As with NPCs *in vivo*, NPCs in low CHIR culture exhibited abundant Six2 indicative of the self-renewal state (Kobayashi et al., 2008; Self et al., 2006) (Fig. 1B,C). In the kidney, *Six2* is downregulated in conjunction with the upregulation of Lef1, a canonical Wnt target and transcriptional effector of Wnt signaling, and the Notch ligand Jag1, a crucial regulator of early nephron patterning (Fig. 1B). In the developing kidney, Lef1^+^/Jag1^+^ cells initially aggregate, then undergo a mesenchymal-to-epithelial transition to establish an epithelial renal vesicle (RV), the anlagen for an adult nephron. Similarly, elevating CHIR levels (5 µM; hereafter, high CHIR) in NPEM induced the differentiation of NPCs, as evidenced by upregulation of Lef1 and Jag1, and the formation of tight aggregates of induced NPCs (Fig. 1C).

To examine CHIR-mediated transcriptional responses, we performed a time course (0-12 h) and assayed key NPC self-renewal genes (*Six2*, *Cited1* and *Eya1*) and differentiation genes (*Wnt4*, *Lef1*, *Jag1*, *Fgf8* and *Lhx1*) by quantitative polymerase chain reaction (qPCR; Fig. S1A). NPCs cultured without CHIR maintained, and elevated, self-renewal-associated markers (Fig. S1A; Guo et al., 2021). In contrast, high CHIR led to a marked and rapid (within 3 h) downregulation in the expression of self-renewal cell markers and enhanced expression of genes linked to the induction of NPCs (Fig. S1A) at both low (37,500 cells/well) or high (300,000 cells/well) seeding densities (Fig. S1B). A marked accumulation of β-catenin was observed in the cell aggregates formed in high CHIR (Fig. S2A). Assessing the purity of isolation using a transgenic reporter demonstrated over 92% of cultured cells showed NPC linked reporter expression, confirming that measured responses reflected NPC gene activity (Fig. S1C).

To confirm low and high CHIR Wnt-pathway associated activity, NPCs were cultured with a bi-specific antibody (BSAB) that mimics Wnt ligands engaging Frizzled and LRP in the Wnt-receptor complex (Janda et al., 2017). NPCs cultured in 500 pM BSAB showed significantly elevated cell proliferation (*P*<0.0001), as measured by DNA incorporation of 5-ethynyl-2′-deoxyuridine (EdU), but no evidence of an inductive response (Fig. S1D,E). In contrast, addition of 4 nM BSAB resulted in a marked downregulation of the NPC marker *Cited1*, and elevated expression of *Jag1* and *Wnt4*, similar to high CHIR conditions (Fig. S1D); the kinetics of *Six2* downregulation differed from high CHIR (Fig. S1D). Collectively, these results indicate that CHIR replicates key features of Wnt receptor activation in NPC programs (Fig. S1D,E).

To examine global CHIR-mediated responses in more detail, bulk RNA-seq profiling was performed on NPC cultures. Comparing high CHIR with low CHIR showed a marked downregulation of genes encoding regulatory factors and genes specific to the NPC self-renewal state (Fig. 1D; Table S1.1) and the activation of genes associated with NPC differentiation (Fig. 1D; Table S1.1). Differential gene expression analysis identified 824 DEGs significantly upregulated and 1205 DEGs significantly downregulated on low to high CHIR transition (Table S1.1). Removal of CHIR (no CHIR) resulted in the upregulation of 603 genes and downregulation of 384 genes relative to low CHIR conditions (Table S1.2). Consistent with biological processes at play, Gene Ontology GO terms highlight ‘nephron epithelium development’ and ‘kidney epithelium development’ as significantly enriched terms for genes differentially expressed when comparing low with high CHIR conditions (Fig. 1E; Tables S1.3 and S1.4). Consistent with the nephrogenic program, terms such as ‘urogenital system development’, ‘renal system development’ and ‘epithelial tube morphogenesis’ are among the top 10 most significant terms. GO term analysis of NPC profiles comparing low and no CHIR highlighted ‘cytoskeletal organization’ and ‘attenuated cell movement’ (elevated in no CHIR; Table S1.5) and ‘cell proliferation’ (elevated in low CHIR; Table S1.6), as reported previously (Guo et al., 2021). Interestingly, in the absence of CHIR, transcript levels were elevated for several transcriptional determinants associated with the NPC self-renewal state (e.g. *Osr1*, *Meox1*, *Meox2* and *Cited1*), consistent with earlier qPCR analysis (Fig. S1A; Table S1.2). Thus, low levels of Wnt/β-catenin signaling attenuate NPC-associated gene activity.

To determine whether the primary inductive response requires new protein synthesis, cycloheximide (CHX), an inhibitor of eukaryotic translation elongation (Burgers and Fürst, 2021; Schneider-Poetsch et al., 2010; Siegel and Sisler, 1963), was added in conjunction with a low to high CHIR switch of NPC cultures. Differentiation association genes were upregulated, albeit to lower levels than high CHIR alone, and the absence of Jag1 synthesis confirmed effective translational inhibition (Fig. S2B-D). Interestingly, CHX treatment inhibited the downregulation of *Six2*, *Cited1* and Eya1, key genes associated with the naïve NPCs (Fig. S2B-D), consistent with a transcriptional and/or translational requirement to exit the NPC program (Fig. S2B-D). Retention of the NPC program in CHX may factor into a weaker inductive response.

### β-Catenin promotes NPC proliferation but not the transcriptional program of self-renewing NPCs

**Fig. 2.**
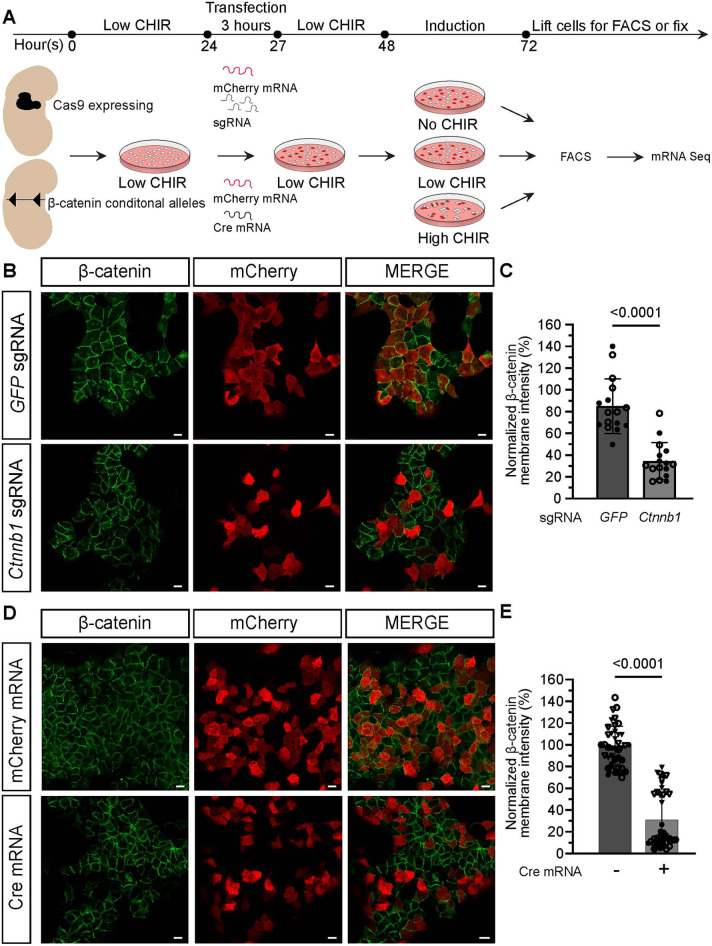
**mRNA transfection provides rapid Cre- or Cas9-mediated removal of β-catenin in primary mouse nephron progenitors.** (A) Schematic of Cre- and Cas9-mediated transfection KO of nephron progenitor cells (NPCs) from Cas9-GFP expressing and β-catenin conditional allele mice. NPCs were isolated, stabilized in low CHIR overnight (18-24 h), transfected with either sgRNAs or Cre mRNA, and mCherry mRNA, and incubated in OPTI-mem for 3 h. NPCs were then cultured in low CHIR for a 24 h KO period before changing to nephron progenitor expansion medium (NPEM) with differing CHIR levels. NPCs were assayed for bulk RNA-seq as well as immunostaining 24 h later. (B) Immunostaining of NPCs 24 h after Cas9-mediated KO before the media change into NPEM with differing levels of CHIR. β-Catenin is in green and mCherry (denoting transfected cells) is in red. Scale bars: 10 μm. (C) Quantification of the reduction in membrane β-catenin protein signal 24 h after Cas9 KO in transfected (mCherry^+^) NPCs (*n*=2 technical replicates, no biological replicates; eight fields of view/well; Mann–Whitney test). Data are mean±s.d. (D) Immunostaining of NPCs 24 h after Cre-mediated KO before media change into NPEM with differing levels of CHIR. β-Catenin is in green and mCherry (denoting transfected cells) is in red. Scale bars: 10 μm. (E) Quantification of the reduction in membrane β-catenin protein signal 24 h after the Cre KO period in transfected (mCherry^+^) NPCs (*n*=2 biological replicates; two or three technical replicates, 8-10 fields of view/well; Mann–Whitney test). Data are mean±s.d.

To examine the role of Wnt-directed transcriptional mechanisms in NPCs, we developed and validated a novel platform for rapid, genetic modification of NPCs by mRNA lipofection (Fig. 2A). Lipofectamine (OPTI-mem) transfection of NPCs with polyadenylated mRNA encoding a GFP reporter showed a rapid onset of protein production: ∼30% of cells exhibited GFP fluorescence 3 h after transfection (Fig. S3A) and normal responses to varying levels of CHIR (Fig. S3B). When mRNAs encoding GFP and mCherry were co-transfected, up to 80% of reporter positive cells co-labeled within 24 h (Fig. S3C,D), highlighting efficient co-transfection of transfection competent NPCs. To characterize gene-knockdown efficiency, NPCs were harvested from mice constitutively expressing a Cas9-GFP allele and transfected with a gRNA targeting GFP and an mRNA encoding mCherry. Fluorescence activated cell sorting (FACS) analysis identified a 75% reduction in GFP signal localized to mCherry^+^ NPCs 24 h post-transfection (Fig. S3E), highlighting effective CAS9 gene editing of transfected cells.

To gain a deeper understanding of the role Wnt/β-catenin signaling plays in the self-renewal and differentiation of NPCs, we used two different mRNA directed approaches to knock out the gene encoding β-catenin (*Ctnnb1*) (Fig. 2A): (1) knockout (KO) of *Ctnnb1* through targeted gRNA transfection into NPCs constitutively producing Cas9 and GFP (Platt et al., 2014); and (2) KO through Cre mRNA-mediated removal in NPCs homozygous for a conditional allele of *Ctnnb1* (Brault et al., 2001). Co-introduction of an mCherry reporter mRNA was used to distinguish transfected cells and a gRNA targeting GFP was used as a control in Cas9-directed KO studies.

**Fig. 3.**
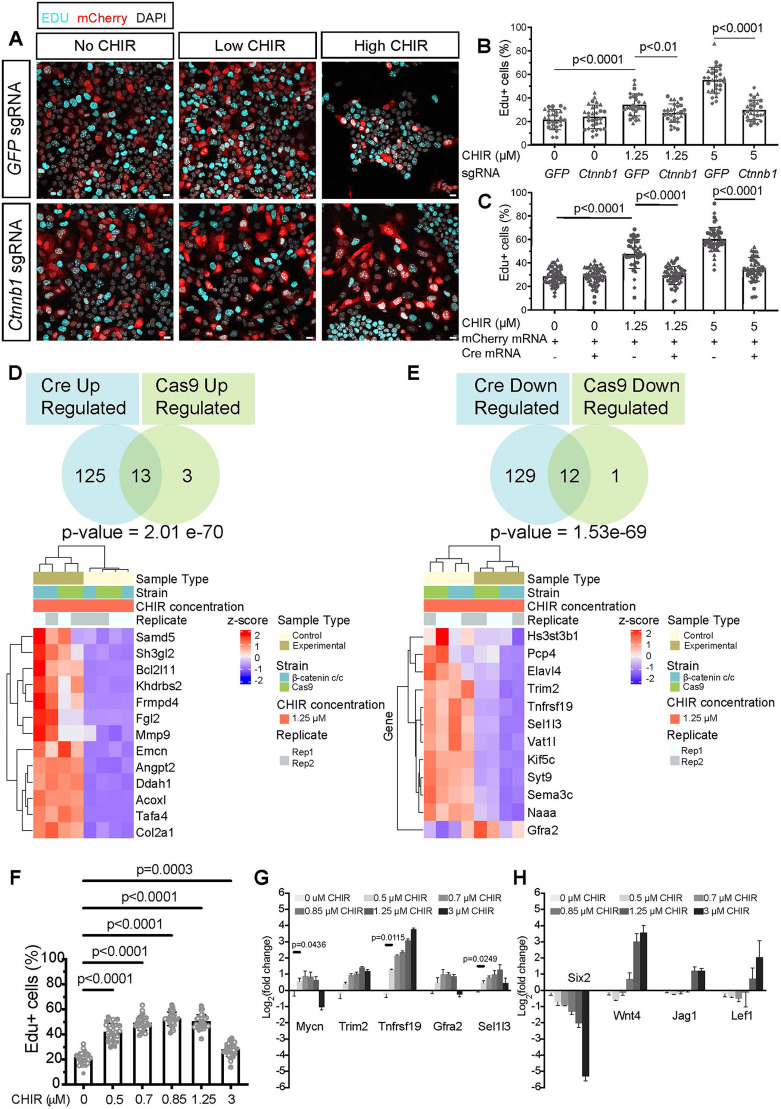
**β-Catenin promotes nephron progenitor cell proliferation but is not a transcriptional regulator of the self-renewal program.** (A) EdU chasing of nephron progenitor cells (NPCs) with Cas9-mediated β-catenin removal after 24 h of culture. Scale bars: 10 μm. (B) Quantification of EdU^+^ cells (proliferating cells) per total number of NPCs in Cas9 β-catenin KO samples (9 or 10 fields of view/coverslip, three biological replicates, unpaired *t*-test). Data are mean±s.d. (C) Quantification of EdU^+^ cells (proliferating cells) per total number of NPCs in Cre β-catenin KO samples for three biological replicates, 7-10 fields of view/coverslip, five technical replicates (except 1.25 control, *n*=4), from left to right: Mann–Whitney-test twice, unpaired *t*-test. Data are mean±s.d. (D) Intersection of DEGs upregulated (Cre up and Cas9 up) from NPCs with β-catenin removed in low CHIR. DEGs calculated with DESEQ2 using with *P*-adjusted cut-offs=0.05 and Log2FC cut-off=0.5. Heatmap of unbiased ranking based on significance of all upregulated genes compared with control low CHIR samples of the intersection of both Cre- and Cas9-mediated removal of β-catenin in low CHIR. (E) Intersection of DEGs downregulated (Cre down and Cas9 down) from NPCs with β-catenin removed in low CHIR. DEGs calculated with DESEQ2 using with *P*-adjusted cut-offs=0.05 and Log2FC cut-off=0.5. Heatmap of unbiased ranking based on significance of all downregulated genes compared with control low CHIR samples of the intersection of both Cre- and Cas9-mediated removal of β-catenin in low CHIR. (F) Dose-response analysis of NPCs with CHIR levels quantifying NPC proliferation by EdU incorporation. Three technical replicates, 10 fields of view/coverslip, D'Agostino-Pearson test. Ordinary one-way ANOVA (*P*<0.05 was considered significant). Data are mean±s.d. (G) Relative transcript levels of *Mycn*, *Trim2*, *Selil3* and *Tnfrsf19* using RT-qPCR analysis in NPCs. Three technical replicates, two-way ANOVA (*P*<0.05 was considered significant). Data are mean±s.d. (H) RT-qPCR of high CHIR-associated targets *Wnt4*, *Jag1* and *Lef1* with new CHIR titers (0.85-1.25 µM). Three technical replicates. Data are mean±s.d.

Genetically appropriate NPCs were isolated from E16.5 kidneys, stabilized in low CHIR overnight and transfected for 3 h in OPTI-mem (Fig. 2A). To enable genetic removal and turnover of mRNA/proteins, NPCs were cultured in low CHIR for 24 h, then cultured for an additional 24 h in either low or high CHIR. Immunostaining showed β-catenin levels were reduced significantly in both gRNA (∼65%) and CRE (∼70%) KO conditions (Fig. 2B-E). Notably, on removal of β-catenin in either low or high CHIR, NPCs showed a marked reduction in EdU assayed proliferation, resembling NPCs cultured without CHIR, demonstrating that β-catenin activity promotes NPC proliferation (Fig. 3A-C; Fig. S1E; Guo et al., 2021). To identify gene-sets enriched in each condition, bulk RNA-seq was performed on reporter positive KO and control cells (Tables S1.7 and S1.8) and the data intersected (Tables S1.9 and S1.10). A set of DEGs were shared between the two KO approaches (Tables S1.9 and S1.10). In low CHIR progenitor maintenance conditions, 13 genes were upregulated and were 12 downregulated on β-catenin removal; no significant alteration was observed in either the NPC transcriptional or proliferative transcriptional signatures (Fig. 3D,E; Tables S1.9 and S1.10). No kidney-relevant GO terms associated with the downregulated gene-set (Table S1.11). However, GO term analysis of genes increased upon Cre and Cas9 β-catenin removal in low CHIR identified a list of 12 terms with kidney development (*Mmp9*, *Bcl2l11* and *Angpt2*) among the enriched terms reflecting an elevation of specific progenitor gene expression, as observed on CHIR removal (Table S1.12).

The 12 shared genes downregulated in both β-catenin KO approaches compared with low CHIR cultured NPCs were intersected with β-catenin/Tcf7/Lef1 ChIP-seq data from NPCs cultured in low and high CHIR (Tables S1.9 and S2; Guo et al., 2021). Although none of the 12 genes showed detectable β-catenin association in low CHIR, eight of these (*Kif5c*, *Trim2*, *Elavl4*, *Sel1l3*, *Vat1l*, *Tnfrsf19*, *Syt9* and *Hs3st3b1*) showed β-catenin association within 200 kb of the transcriptional start site in high CHIR (Table S1.9). Furthermore, for five out of the eight, one or more of the regions corresponded to Encode-predicted cis-regulatory enhancer elements (CREs). Of the four Lef/Tcf DNA-binding factors, Lef1 and Tcf7 most commonly activate transcription, Tcf7l1 represses transcription, and Tcf7l2 has both repressor and activator properties (Lien and Fuchs, 2014). Seven of the eight genes with high CHIR-associated β-catenin showed Tcf7l2 bound at putative CREs in low CHIR, five of these seven genes were also bound by Tcf7l1 in low CHIR. In high CHIR, five showed continuing Tcf7l2 binding, while none retained Tcf7l1 binding. Examining Tcf7 and Lef1 showed no association with DEGs in low CHIR, but six out of the eight β-catenin targeted genes were bound by Tcf7 and/or Lef1in high CHIR (Table S1.9). Together, these data support the engagement of canonical Wnt transcriptional complexes at likely CREs around genes dependent on β-catenin for elevated expression in low CHIR.

Given the stronger response observed on CRE-mediated removal of β-catenin activity versus Cas9-directed mutation (280 versus 29 genes showing significant downregulation; Tables S1.7 and S1.8), we identified the intersection of the CRE removal and low CHIR-dependent gene sets. Consistent with a strong cell cycle signature, ToppGene (Chen et al., 2009) annotated 40% of the gene set (33 of 81) to the cell cycle (Tables S1.13 and S1.14). The 48 shared genes not annotated to the cell cycle (Table S1.13) identified a strong co-expression signature with NPCs in the postnatal mouse kidney in ToppGene analysis (*P*-value 1.4E-20). At this time, the last wave of NPCs showed a low-level activation of the early nephrogenic program (Brunskill et al., 2011), suggesting NPCs in low CHIR culture were in a similar state.

Comparing the cell cycle enriched set with cell cycle annotated genes from no and low CHIR comparison identified only six overlapping genes (*Ska3*, *Ncaph*, *Sgo1*, *Cdc45*, *Brca1* and *Ect2*; Table S1.15). Chromatin association studies in low CHIR showed no interaction at these gene loci of β-catenin or Lef/Tcf factors (Table S2; Guo et al., 2021). Thus, it is likely that none are direct targets of canonical transcriptional complexes. Inspection of the non-cell cycle annotated genes identified *Mycn* (Table S1.13). Mycn and Myc regulate the cell cycle. Further, *Myc* is a well-documented Wnt target (Das et al., 2023; Koelman et al., 2022). Interestingly, chromatin analysis of NPCs in low CHIR identified shared β-catenin and Lef/Tcf factor association at ENCODE predicted CREs flanking *Mycn* (Table S1.16). Annotation of cell cycle-related genes dependent on β-catenin showed a marked G2-M signature consistent with β-catenin removal arresting cycle progression at this stage (Whitfield et al., 2002; Table S1.14).

Next, we performed a dose-response analysis of NPCs to varying CHIR levels quantifying NPC proliferation by EdU incorporation (Fig. 3F) and the relative transcript levels of low (*Mycn*, *Trim2*, *Sel1l3* and *Tnfrsf19*; Fig. 3G) and high (*Wnt4*, *Jag1* and *Lef1*; Fig. 3H) CHIR responsive gene sets. Although variability associated with a new batch of CHIR led to a different dose response (see Materials and Methods), we observed a statistically significant increase in cell proliferation at the lowest CHIR dose (0.5 µM; Fig. 3F) coupled with a statistically significant upregulation of *Mycn*, *Tnfrsf19* and *Sel1l3* (Fig. 3G) but no upregulation of high CHIR-associated targets *Wnt4*, *Jag1* and *Lef1* until the addition of significantly higher CHIR levels (= or >0.85 µM: Fig. 3H). These data, together with β-catenin/Lef/Tcf CRE intersection data, are consistent with different levels of Wnt signaling input translating into distinct transcriptional outputs directed by canonical Wnt transcriptional complexes. These studies highlight a low threshold gene set linked to proliferating NPCs. Elevating CHIR levels resulted in a marked decrease in *Six2* mRNA, suggesting mechanisms to antagonize the NPC state.

### β-Catenin is not essential for NPC progenitor identity but is essential for NPCs to exit the NPC state

**Fig. 4.**
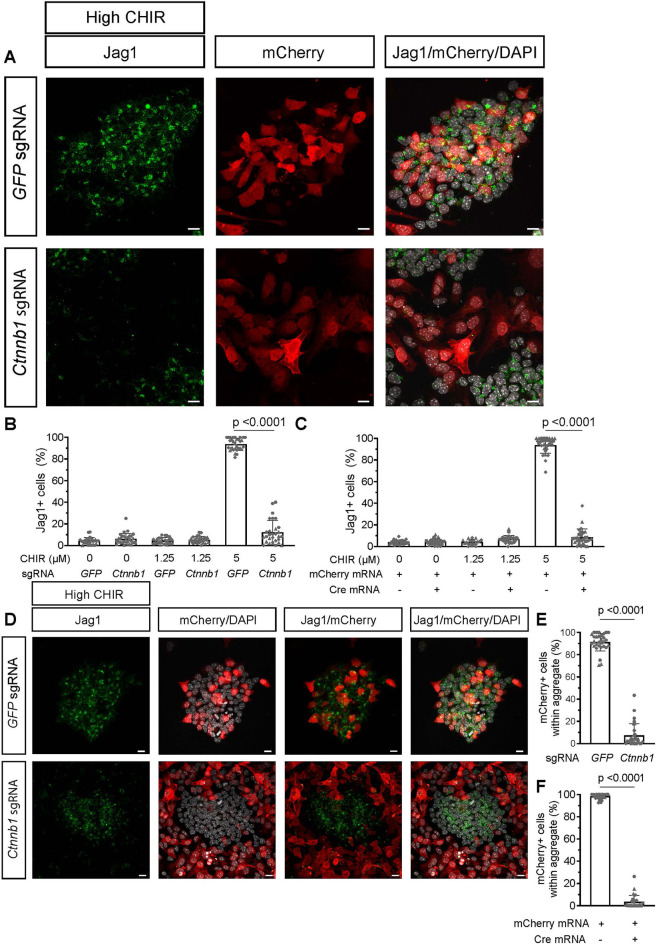
**Induction status and cell behavior changes resulting from β-catenin removal from nephron progenitors in high-CHIR conditions.** (A) Jag1 protein is lost after Cas9-mediated removal of β-catenin in nephron progenitor cells (NPCs) in mCherry^+^ transfected cells in high CHIR. DAPI (white) indicates nuclei. (B) Quantification of Jag1 protein in mCherry^+^ NPCs after Cas9-mediated removal of β-catenin. Seven to 10 fields of view/coverslip, three biological replicates, data are not normally distributed, Mann–Whitney test. Data are mean±s.d. (C) Quantification of Jag1 protein in mCherry^+^ NPCs after Cre-mediated removal of β-catenin. Nine or 10 fields of view/coverslip, two biological replicates, data are not normally distributed, Mann–Whitney test. Data are mean±s.d. (D) NPCs with β-catenin removed (transfected cells) do not form tight aggregates. mCherry marks transfected cells, Jag1 is an induction marker and DAPI (white) indicates nuclei. (E) Percentage of mCherry^+^ transfected NPCs in NPCs with β-catenin removed (Cas9) within aggregates. Ten fields of view/three biological replicates. Data are not normally distributed, Mann–Whitney test. Data are mean±s.d. (F) Percentage of mCherry^+^ transfected NPCs in NPCs with β-catenin removed (Cre) within aggregates. Nine or 10 fields of view/two biological replicates, data are not normally distributed, Mann–Whitney test. Data are mean±s.d. Scale bars: 10 µm.

Elevating CHIR levels resulted in a marked decrease in *Six2* mRNA (Fig. 3H), suggesting increased canonical Wnt signaling activates mechanisms to antagonize the NPC state. Consistent with this view, β-catenin KO in high CHIR resulted in the persistence of Six2, and the repressive Lef/Tcf regulatory factor Tcf7l1, both of which are normally downregulated on induction of NPCs (Guo et al., 2021), as well as a failure to induce Jag1 and aggregate NPCs (Fig. 4A-E; Fig. S4A-F). In short, on β-catenin removal, NPCs cultured in high CHIR resembled NPCs cultured without CHIR (Das et al., 2023; Pan et al., 2017).

**Fig. 5.**
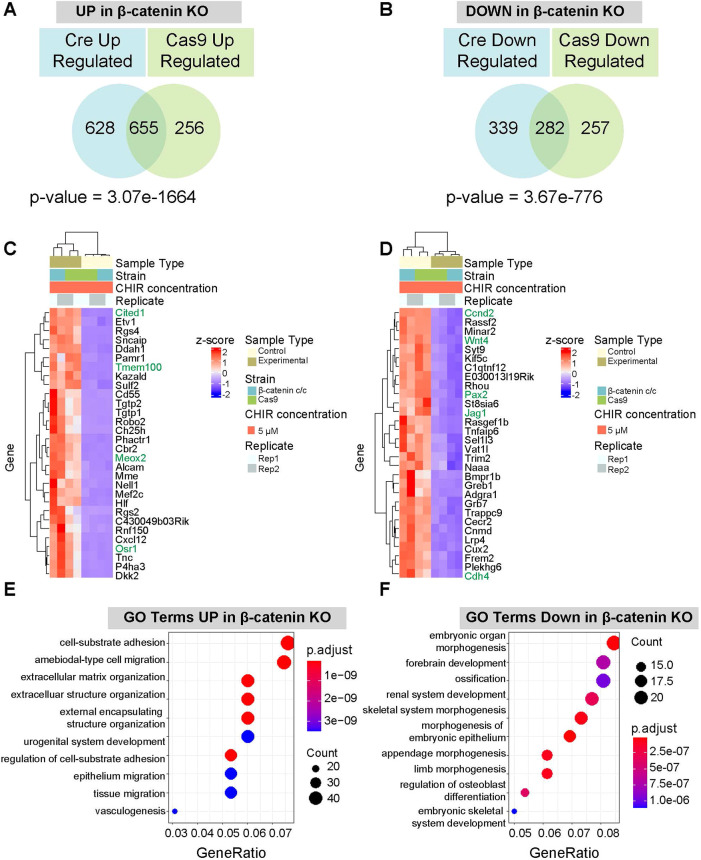
**Transcriptional changes resulting from β-catenin removal in nephron progenitors.** (A) Intersection of DEGs upregulated from Cre- and Cas9-mediated β-catenin removal. (B) Intersection of DEGs downregulated from Cre- and Cas9-mediated β-catenin removal. (C,D) Heatmap of unbiased ranking of top 30 significant upregulated (C) and downregulated (D) genes compared with control samples at the intersection of both Cre- and Cas9-mediated removal of β-catenin in high CHIR. Genes in green are known nephron progenitor cell (NPC) self-renewal markers (C) or known NPC differentiation markers or canonical Wnt/β-catenin targets (D). Genes in green are previously identified markers of either self-renewal (C) or differentiation/canonical Wnt target (D). (E) Top 10 most significant GO terms associated with genes upregulated by β-catenin removal in high CHIR with intersected Cre- and Cas9-meditated DEGs. (F) Top 10 most significant GO terms associated with genes downregulated by β-catenin removal in high CHIR with intersected Cre- and Cas9-meditated DEGs.

Examining DEGs shared between both β-catenin removal conditions in high CHIR cultured NPCs identified a highly significant response (hypergeometric test: *P*-value for upregulated, 3.07E-1664; *P*-value downregulated, 3.675e-776) with 937 shared DEGs: 655 upregulated and 282 downregulated after β-catenin KO, many of which exhibited β-catenin/Tcf7/Lef1 ChIP seq binding associated with the gene locus (Fig. 5A,B; Tables S1.17-S1.20; Table S2). Ranked intersections of shared genes upregulated on KO in high CHIR identified *Cited1* as the most significant gene (*P*=3.57E-36; Log2FC=5.01) together with other well-known markers of an uninduced, self-renewing NPC state, including *Tmem100*, *Meox2* and *Osr1* in the top 30 ranked gene set (Fig. 5C; Table S1.19). NPC self-renewal genes accounted for much of the highly ranked GO term ‘urogenital system development’ recovered from GO analysis of the entire gene set (Fig. 5E; Table S1.21). Other terms such as ‘ameboidal cell movement’, ‘epithelium migration’ and ‘tissue migration’ likely reflect altered cell behaviors observed on induction of NPCs after β-catenin removal (Fig. 5E; Table S1.21).

### β-Catenin transcriptional targets in the induction of nephron progenitors

Among the top 30 most significant downregulated genes on KO of β-catenin in high CHIR were well-characterized genes associated with the differentiation of NPCs, including *Wnt4* and *Jag1* (Fig. 5D), and the cell cycle regulator *Ccnd2* (Fig. 5D; Table S1.19). As expected, top GO terms in this group of genes were associated with: kidney developmental programs: ‘embryonic organ morphogenesis’, ‘renal system development’ and ‘morphogenesis of an embryonic epithelium’; and Wnt signaling, including ‘Wnt signaling pathway’ and ‘cell-cell signaling by Wnt’, consistent with a modified Wnt-signaling response (Fig. 5E,F; Table S1.22).

**Fig. 6.**
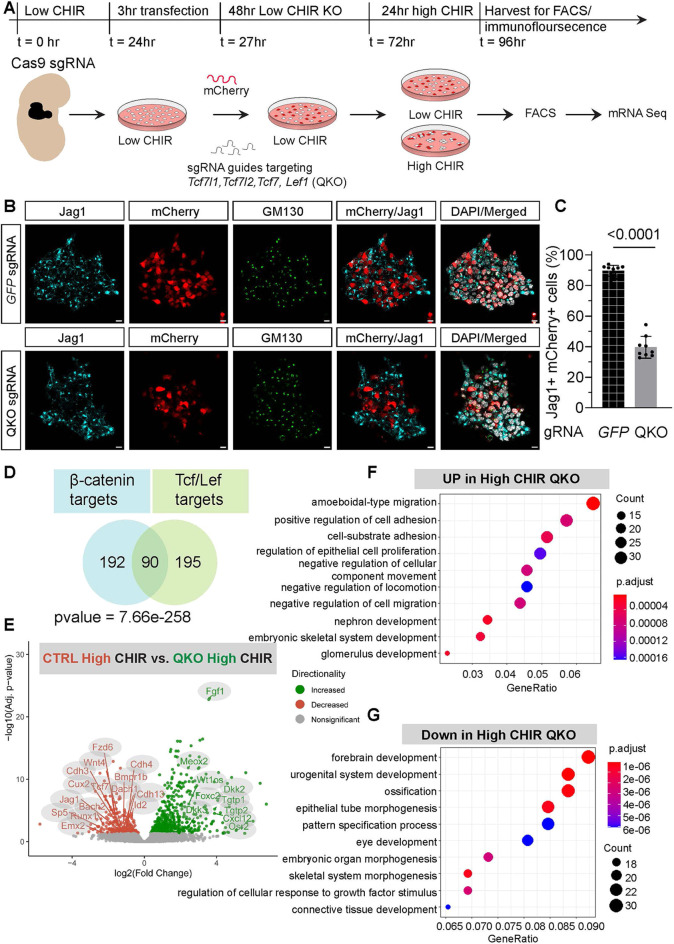
**Tcf/Lef transcription factor removal in nephron progenitors enables β-catenin target validation.** (A) Schematic of Cas9 mediated removal of Tcf7l1, Tcf7l2, Tcf7 and Lef1 in nephron progenitor cells (NPCs) from Cas9-GFP-expressing mice. NPCs were isolated, stabilized in low CHIR overnight (18-24 h), transfected with gRNAs (Tcf7l1, Tcf7l2, Tcf7 and Lef1 in experimental conditions or GFP gRNA in CTRL) along with mCherry mRNA and incubated in OPTI-mem for 3 h. NPCs were then cultured in low CHIR for a 48 h KO before changing for nephron progenitor expansion medium (NPEM) with differing CHIR levels. NPCs were assayed for mRNA-seq and immunostaining 24 h later. (B) Immunofluorescence staining of NPCs for Tcf7l1, Tcf7l2, Tcf7 and Lef1 (QKO) and control (GFP sgRNA). Induction marker Jag1 is in cyan; mCherry is in red; Golgi are marked using GM130 antibody (green); DAPI is in gray. Scale bars: 10 μm. (C) Quantification of the induction marker Jag1 in QKO NPCs cultured in high CHIR. Unpaired *t*-test. One biological replicate, eight fields of view. Data are mean±s.d. (D) Intersection of QKO target genes (genes lost in QKO with Log2FC cut-off>0.5 and *P*-adj cut-off<0.05) with Ctnnb1 KO target genes (genes lost in Ctnnb1 Cre and Cas9 KO with Log2FC cut-off>0.5 and *P*-adj cut-offcut-off<0.05). (E) Volcano plot of DEGs comparing QKO samples with control in high CHIR conditions. (F) Top 10 most significant GO terms of DEGs upregulated in QKO samples in high CHIR conditions. (G) Top 10 most significant GO terms of DEGs downregulated in QKO samples in high CHIR conditions.

Fluorescent *in situ* hybridization and mouse single cell RNA sequencing (scRNA-seq) data analyses were used to characterize transcripts encoding each of the DNA-binding components of canonical Wnt transcriptional complexes. *Tcf7l1* expression was highest in self-renewing NPCs, *Lef1* expression was highest after NPC induction, while *Tcf7* and *Tcf7l2* expression did not change markedly when comparing uninduced and induced NPCs (Fig. S5A-C; Guo et al., 2021; Kim et al., 2024). To assay the requirement for Tcf/Lef factors in NPC programs, we optimized individual gRNAs for the removal of each factor, extending the culture protocol for an additional 24 h after gRNA introduction (48 h total) to ensure a strong knockdown of Tcf/Lef factors (Fig. 6A). Importantly, 24 h of additional NPC culture did not alter gene expression trends for any of the key anchor genes employed throughout this study (Table S1.23). Individual gRNAs led to a removal of greater than 80% of *Tcf7l1*, *Tc7l2* and *Lef1* and 50% of Tcf7 (Fig. S6A-H). Combining gRNAs to target these four *Lef*/*Tcf* genes together in high CHIR NPC culture resulted in ∼50% reduction of Jag1^+^ cells, using Golgi-localized Jag1 as a cell-autonomous maker of induction (Fig. 6B,C). Thus, KO of *Lef*/*Tcf* genes attenuated the CHIR-directed differentiation response, consistent with β-catenin acting, at least in part, through a canonical Tcf/Lef transcriptional pathway.

Principal component analysis (PCA) of bulk mRNA-sequencing on genetically modified NPCs from the quadruple Lef/Tcf KO population (QKO) in high CHIR demonstrated QKO cells clustered closer to NPCs maintained in low CHIR conditions (Fig. S5D). As expected from β-catenin experiments, DEG analysis identified few differences between wild-type and QKO (*n*=24) samples in low CHIR conditions (Table S1.24). In contrast, a large number of DEGs (*n*=857) distinguished control and QKO NPCs in high CHIR (Fig. 6E). Of these, 572 were upregulated and 285 were downregulated on *Lef/Tcf* removal (Table S1.25). Approximately 30% were shared with the β-catenin removal set in high CHIR conditions (Fig. 6D; Table S1.26) and, as expected, GO terms overlapped with this gene set (Fig. 6E,F; Tables S1.27 and S1.28). Analysis of β-catenin binding from high CHIR ChIP-seq datasets showed a significant overlap with this gene set: 77% (69/90) of genes showed localized β-catenin association (Fisher's exact *t*-test, *P*<2.2e-13; Table S1.26). These data support the conclusion that β-catenin acts through Lef/Tcf factors in regulating the transcriptional inductive response initiating differentiation of NPCs.

**Fig. 7.**
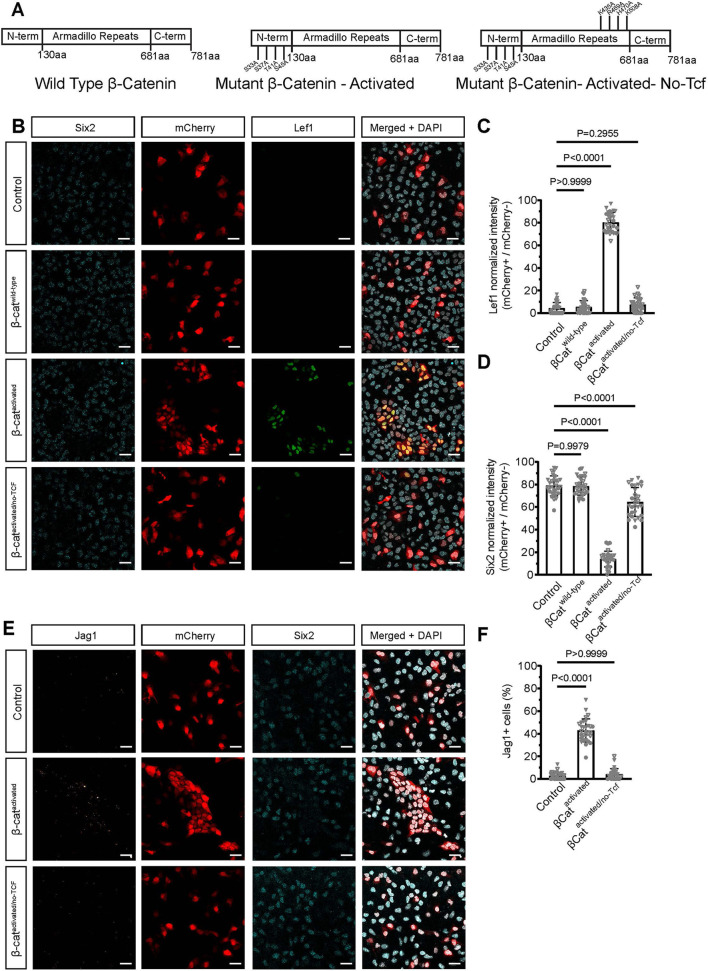
**Overexpression of mutant forms of β-catenin suggest that CHIR-mediated inductive responses of β-catenin act through Lef/Tcf DNA-binding partners.** (A) Schematic of wild-type and mutant forms of mRNA for β-catenin. (B) Immunostaining of NPCs cultured in 0.7 µM CHIR. Post-transfection NPCs were cultured in 0 µM CHIR. Six2 is in cyan; mCherry is in red, Lef1 is in green. Scale bars: 25 µm. (C,D,F) Transduction with βCat^activated^ resulted in a strong cell-autonomous induction of Lef1 (C), the downregulation of Six2 (D) and the induction of Jag1 (F). (C) Two biological replicates and one technical replicate, 10 fields of view/coverslip, D'Agostino-Pearson test. Ordinary one-way ANOVA (*P*<0.05 was considered significant). Data are mean±s.d. (D) Two biological replicates and one technical replicate, 10 fields of view/coverslip, D'Agostino-Pearson test. Ordinary one-way ANOVA (*P*<0.05 was considered significant). Data are mean±s.d. (E) Immunostaining of NPCs cultured in 0.7 µM CHIR. Post-transfection NPCs were cultured in 0 µM CHIR. Jag1 is in orange, mCherry is in red, Six2 is in cyan. Scale bars: 25 µm. (F) Two biological replicates and one technical replicate, 10 fields of view/coverslip, D'Agostino-Pearson test. Ordinary one-way ANOVA (*P*<0.05 was considered significant). Data are mean±s.d.

To examine β-catenin actions directly, we introduced mRNAs encoding FLAG-epitope variants of β-catenin (βCat^wild-type^) into NPCs: an activated form of β-catenin (βCat^activated^) containing four point mutations that abolish GSK3-directed proteasomal degradation of β-catenin (Wu et al., 2009) or an activated form with additional mutations (βCat^activated/no-Tcf^) designed to abolish β-catenin binding to Lef/Tcf factors (Koelman et al., 2022; Fig. 7A). In the absence of CHIR, FLAG immunostaining indicated effective turnover of wild-type β-catenin, whereas the two activated forms of wild-type β-catenin were readily detected in transduced cells (Fig. S7). No induction of Lef1 was observed in NPCs on transduction with βCat^wild-type^ mRNA (Fig. 7B-D). In contrast, transduction with βCat^activated^ resulted in a strong cell-autonomous induction of Lef1 (Fig. 7B,C) and Jag1 (Fig. 7E,F), and the downregulation of Six2 (Fig. 7B,D). All βCat^activated^ mRNA associated responses were markedly reduced when Lef/Tcf interactions were abolished in βCat^activated/no-Tcf^ transduced NPCs (Fig. 7B-F). Collectively, these data support the conclusion that CHIR-mediated inductive responses reflect the actions of β-catenin acting through Lef/Tcf DNA binding partners.

**Fig. 8.**
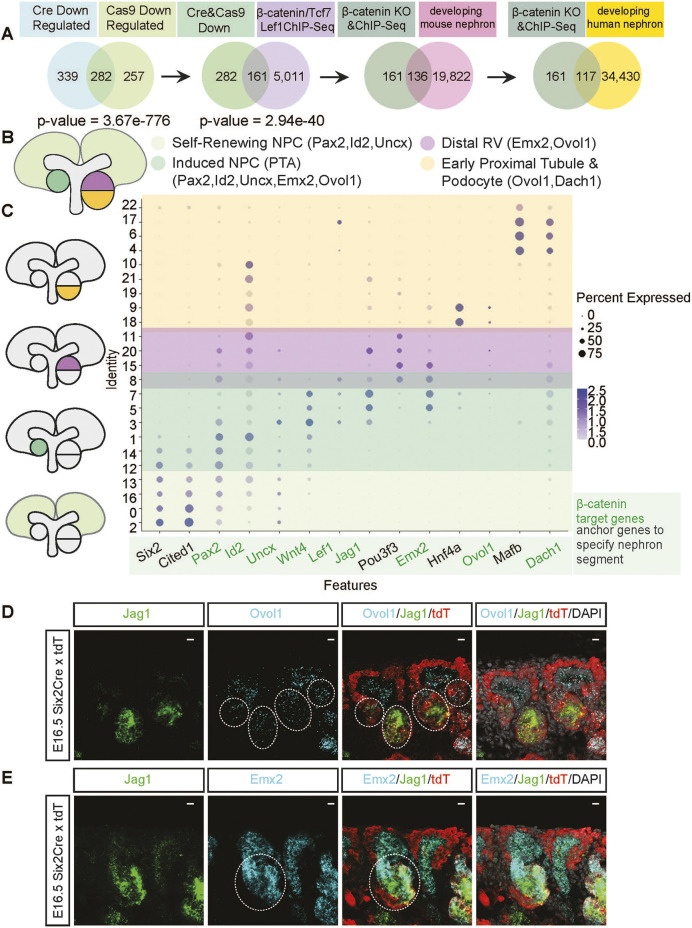
**Integration of ChIP-seq data and scRNA-seq data with β-catenin target genes reveals genes responsible for the induction of NPCs and patterning of the developing nephron.** (A) Intersection of β-catenin target genes with β-catenin/Tcf7/Lef1 ChIP-seq binding and integration with scRNA-seq data narrows down a significant list of 137 genes expressed in the developing nephron. (B) Schematic of the nephrogenic niche. PTA, pre-tubular aggregate; RV, renal vesicle. (C) Dot plot denoting co-expression of β-catenin target genes *Pax2*, *Id2*, *Uncx*, *Emx2*, *Ovol1* and *Dach1* with known markers of self-renewing NPCs (*Six2* and *Cited1*), an early induction gene (*Wnt4*), a distal marker (*Pou3f3*), a proximal tubule marker (*Hnf4a*) and a podocyte gene (*Mafb*) in mouse p0 scRNA-seq. (D) RNA-scope of *Ovol1* on E16.5 Six2TGCxTdt kidneys. Scale bars: 10 μm. Dotted lines outline Ovol1 transcripts in differentiating NPCs. (E) RNA-scope of *Emx2* on E16.5 Six2TGCxTdt kidneys. Scale bars: 10 μm. Dotted lines outline *Emx2* transcripts in differentiating NPCs.

To predict Wnt/β-catenin targets within the NPC inductive response, we intersected the 282 β-catenin-dependent genes from the dual genetic KO gene list with published ChIP-seq analysis of the chromatin association of β-catenin, Tcf7 or Lef1 in high CHIR conditions (*n*=5011 associations; Table S2; Guo et al., 2021). A significant overlap (*P*=2.94e-40) was observed with 161 genes using HOMER (Duttke et al., 2019) to assign genes to peaks within 100 kb of the transcriptional start site (Table S1.29). Intersecting these putative target genes with annotated scRNA-seq datasets of the developing (p0) mouse kidney undergoing early patterning from NPCs identified 137 of the 162 genes within developing mouse nephron *in vivo* (Kim et al., 2024) (Table S1.30). Examining this set in parallel data for the developing human kidney (Lindström et al., 2021) showed the majority (117) were expressed within similar populations in the developing human kidney (Fig. 8A; Table S1.31). These analyses provide strong evidence that *in vitro* inductive responses in CHIR-treated NPCs reflect *in vivo* activity of β-catenin driven, and Lef/Tcf directed, transcriptional programs in committed NPC cell types.

β-Catenin target genes (β-catenin and Tcf/Lef factor binding in high CHIR conditions) included *Ovol1* and *Emx2* (Fig. S8D,E), two transcriptional regulators with potential roles in nephron patterning and linked to kidney cysts and urogenital defects, respectively, in mutants (Miyamoto et al., 1997; Teng et al., 2007). Fluorescent *in situ* hybridization demonstrated that *Ovol1* and *Emx2* were expressed early in differentiating NPCs, overlapping previously established NPC differentiation markers genes (Fig. 8B-E). Subsequently, *Ovol1* was expressed broadly within the developing renal vesicle, whereas *Emx2* was enriched in distal nephron regions (Fig. 8B-E). To examine *Ovol1* function, we generated mice carrying homozygous null mutations, but could not replicate previously reported cystic kidney phenotypes.

## DISCUSSION

Our analysis of β-catenin action in the self-renewal programs that lead to NPC expansion and the inductive program that initiates the process of nephrogenesis leads to several broad conclusions.

(1) Wnt/β-catenin activity does not maintain NPC identity. Indeed, the NPC signature of key transcriptional components, including *Six2*, are enhanced on removal of either CHIR or β-catenin. Analysis of the high CHIR inductive response indicates a cycloheximide-dependent process attenuating *Six2* mRNA levels. The undefined mechanism is an interesting target for future studies.
(2) Both low CHIR and β-catenin promote expansion of NPCs in NPEM *in vitro*, in line with ectopic Wnt activation within NPCs (Ramalingam et al., 2018). Interestingly, removal of β-catenin results in pronounced cell-cycle transcriptional signature that is consistent with cell cycle arrest at the G2-M transition, although direct analysis of cell cycle parameters are required to give a conclusive insight. Chromatin analyses do not support a direct interaction of canonical Wnt transcriptional complexes with core cell cycle genes. However, mRNA-seq analysis identified *Mycn* as a target of low CHIR, β-catenin-dependent regulation (CRE knockdown dataset), and chromatin analysis indicated a direct association between β-catenin and canonical Wnt transcription factors at ENCODE predicted CREs at the *Mycn* locus in low CHIR cultured NPCs. Although there is no strong evidence in the literature identifying *Mycn* as a target of the canonical Wnt pathway, Myc is a well-documented mediator of Wnt-directed growth responses in a number of normal and oncogenic contexts (Das et al., 2023; Koelman et al., 2022). Interestingly, *Myc* and *Mycn* removal from mouse NPCs *in vivo* shows an overlapping role for each in the expansion of NPCs, consistent with Mycn being a direct mediator of β-catenin-dependent NPC expansion *in vitro* (Pan et al., 2017). The observation that quiescence correlates with elevated levels of Six2 suggests NPC replication reduces steady-state levels of this and potentially other key NPC determinants.
(3) Collectively, experiments removing β-catenin in the presence of low or high CHIR, or adding stabilized mutated forms of β-catenin in the absence of CHIR, point to CHIR-directed dose-dependent canonical Wnt transcriptional responses. Chromatin association with low and high CHIR target genes supports their direct regulation by canonical Wnt transcriptional complexes. Consistent with this view, removing Lef/Tcf factors from NPCs in high CHIR, and attenuating interactions between stabilized β-catenin and Lef/Tcf factors in the absence of CHIR, support a crucial role for canonical Wnt complexes in activating the nephrogenic program. Although these data suggest β-catenin-dependent canonical Wnt transcriptional processes predominate downstream of CHIR stimulation of NPCs, β-catenin has been shown to interact in several other pathways (Li et al., 2023). With a better understanding at the molecular level of β-catenin interactions in non-canonical transcriptional pathways, the NPC model system evaluated here is well suited to rigorously assess potential interactions in NPC programs.
(4) High CHIR invokes a β-catenin-dependent aggregation of NPCs. Furthermore, aggregation is invoked by stabilized forms of β-catenin in the absence of CHIR, but only if these are competent to bind with Lef/Tcf factors. These observations support a dual role for β-catenin in driving transcriptional programs activating nephrogenesis and the cellular programs initiating the mesenchymal-to-epithelial transition of NPCs in the formation of the nephron anlagen, the renal vesicle. The cellular aggregation responses are explored within a companion paper (Der et al., 2024).

### The role of β-catenin in self-renewing nephron progenitors

mRNA-seq analysis of genes showing low CHIR dependent upregulation in NPCs that was dependent on β-catenin, as evidenced by both Cas9 and Cre removal, identified a small set of putative low-threshold canonical Wnt targets. None of these showed a low CHIR β-catenin association. However, assessing the binding of β-catenin to DNA through indirect association with Lef/Tcf factors presents a challenge, likely most acute close to the signaling threshold. Importantly, most of these putative targets associated with β-catenin in high CHIR at ENCODE predicted CREs that were bound by Lef/Tcf factors in both low and high CHIR conditions. These data support direct regulation of low threshold gene targets by canonical Wnt complexes.

Among the canonical Wnt associated β-catenin targets, *Tnfrsf19* is activated by Wnt signaling in colon stem cells, negatively regulating their Wnt responsiveness: a negative-feedback component in canonical Wnt signaling (Fafilek et al., 2013). For non-β-catenin-associated genes, *Gfra2* encodes a receptor for the ligand Gdnf and Ntn (Yoong and Too, 2007). Gdnf secreted by the capping mesenchyme (NPCs and adjacent interstitial progenitor cells) signals through Gfra1/Ret receptor complexes present on underlying ureteric epithelial progenitors of the collecting system, stimulating branching growth of the network (McMahon, 2016). Given an absence of Ret in NPCs, Gfra2 is unlikely to play a direct role in signaling in NPCs but an ability to bind Gdnf could facilitate concentration and/or presentation of this crucial ligand. Finally, *Sema3c* has recently been shown to upregulate Wnt-pathway activity independently of ligand by promoting nuclear accumulation of β-catenin: a positive feed-forward component in canonical Wnt signaling (Hao et al., 2023).

Previous studies directly analyzing mouse kidney explants identified Wnt9b-dependent expression of genes in self-renewing NPCs (Karner et al., 2011), linking Wnt9b to the maintenance of NPC programs and properties (Carroll et al., 2005). Seven of the 16 reported Wnt9b-regulated genes in these studies showed elevated expression of at least one isoform in low CHIR versus no CHIR analyzed in previous reports (Guo et al., 2021). In the data here, identifying minimal levels of CHIR that support expansion of NPCs, while not significantly activating established Wnt targets of the inductive response (*Wnt4* and *Lef1*), found that none of the genes overlapped the low CHIR, Cas9 and Cre removal intersected gene set.

However, *Amph* and *Btbd11* (also known as *Abtb3*), which are classified as progenitor target genes (Karner et al., 2011), and *C1qtnf12* (also known as *C1qdc2*) and *Cdh4*, which are classified as components of the nephrogenic inductive response (Karner et al., 2011), were identified in our data as low CHIR β-catenin-dependent targets in the more-extensive Cre-dependent gene set. Interestingly, upregulation of *Cdh4* is linked to the high CHIR-dependent cell aggregation response in the companion paper, suggesting a low-threshold response to activating *Cdh4* expression (Der et al., 2024). The *Cdh4*-containing gene set includes a number of other genes, such as *Pax2*, *Hey1* and *Sall1*, that are further elevated under high-CHIR conditions, highlighting a differential sensitivity of genes linked to the nephron forming program.

Expansion of both mouse and human nephron progenitors is dependent on low concentrations of CHIR (Brown et al., 2013; Huang et al., 2024; Taguchi et al., 2014). CREs associated with *Myc* and *Mycn* are engaged by β-catenin and Lef/Tcf factors, but only Mycn shows a significant dependence on β-catenin at the cut-off conditions in our analysis, suggesting a predominant role for Mycn in Wnt-directed proliferation of NPCs. *In vivo*, both Mycn and Myc are required for normal expansion of NPCs (Pan et al., 2017). Future studies will benefit from the efficient RNA-directed genetic modification developed here to directly address the significance of Mycn-associated CREs in mediating Wnt-directed stimulation of NPC proliferation. *In vivo* and *in vitro* evidence shows Fgf signaling is also a crucial mediator of NPC proliferation for mouse and human NPCs (Brown et al., 2011, 2013, 2015; Barak et al., 2012; Taguchi et al., 2014; Huh et al., 2020; Huang et al., 2024). How Wnt and Fgf signals coordinate to control NPC proliferation remains to be determined.

### β-Catenin/Tcf/Lef transcriptional targets of the inductive response

Examining the inductive response *in vitro* provides new insight into Wnt/β-catenin targets in the early activation of the nephrogenic program. Many of these intersect with independent studies predicting transcriptional targets through chromatin association studies examining β-catenin and Lef/Tcf factors in NPCs *in vivo* (Park et al., 2012) and *in vitro* in NPEM culture (Guo et al., 2021). Lef/Tcf removal studies, in conjunction with β-catenin KO and gain-of-function β-catenin variants, with or without Lef/Tcf interaction capacity, indicate that β-catenin acting in partnership with Lef/Tcf factors underpins β-catenin-mediated induction of NPCs. Whereas these data substantiate a major role for canonical Wnt transcriptional mechanisms, we cannot rule additional mechanisms of β-catenin-dependent gene regulation.

Upregulation of *Jag1*, *Wnt4* and *Lef1* confirmed expected outcomes for inductive signaling in high CHIR conditions, and RNA-seq identifies an extensive set of putative targets of canonical Wnt activity for future studies. *Cux2*, *Ovol1*, *Sox11*, *Bach2*, *Id2*, *Lef1*, *Tcf7* and *Emx2* are predicted direct transcriptional targets of canonical Wnt complexes in line with data from a variety of other systems (Hasenpusch-Theil et al., 2018; Kormish et al., 2010; Mahmoudi et al., 2009; Piloto and Schilling, 2010; Rockman et al., 2001; Schmidt-Ott et al., 2007). Each of these genes encodes a transcriptional regulator indicative of a complex transcriptional output initiated by a simple switch: elevated levels of nuclear β-catenin. Interestingly, *Emx2* and *Ovol1* are both expressed in the distal epithelial regions of the developing nephron, in both the developing mouse and human nephron anlagen, closest to a continuing source of Wnt9b signal, in Lef1^+^ epithelium. Thus, Wnt responses may encompass both primary inductive outcomes and later polarity-regulating mechanisms in the nephron anlagen.

At the signaling level, we observed a β-catenin-dependent upregulation of genes encoding ligands in Fgf (*Fgfr2*), Notch (*Jag1*), Tgfa (*Tgfa*), Vegf (*Vegfc*), Wnt (*Wnt4*) and Bmp (*Bmp7*, *Bmp2* and *Bmpr1b*) signaling in high CHIR. BMPs have been shown to play an important role in NPC self-renewal and differentiation (Brown et al., 2013; O'Brien, 2019; Oxburgh et al., 2011, 2014). Inductive Wnt/β-catenin signaling is proposed to act upstream of Bmp signaling. *Bmp7* is expressed in RVs overlapping *Jag1* and *Wnt4* (Park et al., 2012). Bmp7 has been proposed to signal through Bmpr1b from analysis of scRNA-seq data from the developing mouse kidney (Combes et al., 2019). *Bmp7* enhancers are bound by β-catenin at predicted Lef/Tcf-binding motifs. Furthermore, *Bmp7* enhancer activity is lost on mutation of Lef/Tcf-binding sites within *cis*-regulatory modules regulating Bmp7 activity in developing nephrons (Park et al., 2012). *Wnt4* is itself an inductive signal, maintaining the initial Wnt9b-triggered response as an autoregulatory signal (Stark et al., 1994) that is transcriptionally regulated by canonical Wnt-regulatory complexes (Park et al., 2012).

In addition to *de novo* induction of genes in high CHIR, β-catenin activity was required to silence expression of genes associated with the NPC self-renewal state, including *Six2* and *Cited1*. Interestingly, cyclohexamide studies suggest that although the early inductive program was activated in high CHIR, there was no reduction in the expression of key genes associated with the NPC state. Thus, new protein synthesis is not essential for inducing key Wnt targets but is essential for silencing the NPC-associated regulatory program. Examining the list of putative β-catenin targets identifies the known transcriptional repressor *Id2*. *Id2* induction in high CHIR is perturbed on loss of β-catenin or QKO of Lef/Tcf factors. Id2 is a mediator of the BMP pathway, suggesting a potential link between Wnt and Bmp signaling (Blank et al., 2009; Brown et al., 2013; Lindström et al., 2015a,b), and is reported to act alongside *Six2* in gut development (Mori et al., 2018). Mechanistically, Wnt/β-catenin targeted activation of an NPC inhibitory factor(s) could provide a rapid feed-forward activity that destabilizes the progenitor state.

### Canonical Wnt regulation in other stem/progenitor systems

Several well characterized developmental processes, and the regenerative capacities of adult stem cell systems, rely on canonical Wnt regulation of stem cell populations (Briscoe and Small, 2015). Hair follicle stem cells (HFSCs) in the skin and intestinal stem cells (ISCs) in the gut (Clevers et al., 2014; Gehart and Clevers, 2019; Lien et al., 2014) are some of the best-studied examples. In HFSCs, Tcf7l1 and Tcf7l2 are present in the quiescent Wnt (inactive or low) state. Canonical Wnt signaling input, ‘activating’ Tcf7 and Lef1 factors, invokes the transition of HFSCs to a highly proliferative state along with the activation of canonical Wnt targets (Guo et al., 2021; Lien and Fuchs, 2014; Merrill et al., 2001). Targeted removal of β-catenin from the HFSC population blocks Wnt-mediated induction of HFSCs (Choi et al., 2013; Lien et al., 2014), much as β-catenin removal from NPCs blocks CHIR-mediated induction of the nephrogenic program *in vitro* and Wnt-directed differentiation in the *in vivo* kidney (Park et al., 2007). Conversely, overexpression of β-catenin enhances ectopic NPC differentiation in the kidney and hair follicle induction in the skin (Gat et al., 1998; Lowry et al., 2005; Närhi et al., 2008; Park et al., 2007). In the mammalian gut, the Wnt pathway has been linked to the maintenance and differentiation of epithelial stem cells (Barker et al., 2007; Gehart and Clevers, 2019; Sato et al., 2011; Yan et al., 2017). Loss of *Ctnnb1* and *Tcf7* functions in the gut leads to a dramatic loss of ISCs (Fevr et al., 2007; Korinek et al., 1998).

The current study highlights a tractable model system replicating key features of renal development that can facilitate an understanding of crucial biological processes at play in development of the mammalian kidney and help to provide a complementary insight into the broader role of Wnt signaling mechanism in stem cell and progenitor cell programs (Gehart and Clevers, 2019; McMahon, 2016; van Es et al., 2012).

## MATERIALS AND METHODS

### Mouse husbandry

All animal use was approved by the University of Southern California IACUC and followed institutional and federal guidelines. Pregnant Swiss Webster or C57Bl/6 mice carrying Cas9, β-catenin c/c and/or Six2-TGC alleles were euthanized to isolate NPCs from E16.5 kidneys. The sex of embryonic kidney donors was not distinguished.

### NPC isolation and culture

NPEM formulation and NPC isolation followed the previously published protocol (Brown et al., 2015; see Table S3). Kidneys were harvested from E16.5 mouse embryos and placed into PBS on ice. Each kidney was expected to yield approximately 150,000 NPCs (100,000 NPCs for B6 background). After dissection, kidneys were washed with 2 ml HBSS (Thermo Fisher Scientific, 14175-095) twice to remove blood and shaken on a Nutator platform for 2 min at 495 rpm, then incubated in 2 ml HBSS solution containing 2.5 mg/ml collagenase A (Roche, 11 088 793 001) and 10 mg/ml pancreatin (Sigma, P1625) for 15 min at 37°C while rocking on a Nutator platform at 495 rpm. The enzymatic reaction was then terminated by the addition of 125 μl of fetal bovine serum (FBS). The resulting supernatant was pelleted then passed through a 40 μm filter, and then washed with AutoMACS running buffer (Miltenyi, 130-091-221) before spinning down at 500 ***g*** for 3 min. The cell pellet, predominantly cells of the cortical nephrogenic zone, was resuspended in 76 μl of AutoMACS running buffer from 10 pairs of kidneys. NPC enrichment resulted from the removal of other cell types in the cell suspension using a combination of the following PE-conjugated antibodies (see Table S3): 9 μl anti-CD105-PE; 9 μl anti-CD140-PE; 8 μl anti-Ter119-PE (Miltenyi, 130-102-893); and 1.6 μl anti-CD326-PE (Miltenyi, 130-118-075).

The cells and antibodies were incubated at 4°C for 30 min without agitation on ice, then washed three times with 1 ml AutoMACS running buffer. To remove the unwanted non-NPCs, 20 μl of anti-PE beads were added to the cell suspension for 30 min on ice. Cells were then washed three times in 1 ml of running buffer, and finally cells resuspended in 1 ml of AutoMACS running buffer and sent through the AutoMACS program, as described in a previously published protocol (Brown et al., 2015) to remove non-NPC cell types enriching for NPCs.

NPCs were seeded at 300,000 cells/well on a 24-well plate. For β-catenin mRNA overexpression experiments, NPCs were seeded at 150,000 cells/well on a 24-well plate. The 24-well NPC culture plates were treated with Matrigel (Corning, 354277) (Matrigel: APEL, 1:25) in APEL medium and incubated at room temperature in cell culture hood for at least 1 h. APEL was then aspirated off leaving behind remaining Matrigel. For all culture experiments NPCs were seeded in low-CHIR NPEM. Upon seeding, plates were shaken three times every 10 min to evenly distribute cells throughout the well. Importantly, after receiving a new batch of CHIR, we observed a markedly higher dose-dependent response. This may be due to some variability at the level of the manufacturer. Therefore, we conducted a titration experiment to identify a novel basal concentration that matched most of the features observed in the bulk of the study, including the absence of upregulation of high-CHIR-associated targets *Wnt4*, *Jag1* and *Lef1*, and a statistically significant elevation of proliferation*.* This concentration 0.7 μM CHIR was used as a basal ‘low CHIR’ concentration in all experiments that included β-catenin mRNA overexpression. Upon transfection, NPCs were cultured in 0 μM CHIR NPEM for 24 h.

### Cycloheximide experiments

Cycloheximide (CHX) (14126, Cayman Chemical) was resuspended in DMSO and stored as 25 mg/ml stock solution at −20°C. To achieve the final 10 μM working concentration, 20 μl stock solution was mixed with 80 µl APEL-2. From this solution 5.6 μl was pipetted to 10 ml high-CHIR NPEM. Cycloheximide was used 10 μM as it has shown efficiency previously in different *in vitro* systems (Burgers and Fürst, 2021; Schneider-Poetsch et al., 2010; Siegel and Sisler, 1963). For vehicle control, a matching DMSO solvent control was used. NPCs were seeded at 300,000 cells/well and CHX treatment was applied for 12 h simultaneously with the high CHIR induction. Cell viability was evaluated using the LIVE/DEAD Viability/Cytotoxicity Kit, for mammalian cells (Thermo Fisher Scientific). Media was then aspirated off and cells were trypsinized. Enzymatic reaction was stopped with autoMACS running buffer+10% FBS. After centrifuging for 3 min at 500 ***g***, we followed the LIVE/DEAD Viability/Cytotoxicity Kit, for mammalian cells (Thermo Fisher Scientific) protocol. Images of cell suspension were acquired by Leica Thunder system with 10×0.45 NA objective with green and red filter cubes on 24-well cell culture plates. The percentage of dead/live cells was quantified and calculated by Imaris using spot module.

### *In vitro* mRNA synthesis

Cre mRNA was created using pCAG-Cre plasmid (Addgene, 13775), mCherry mRNA was created using (Addgene pX330-U6-Chimeric_BB-CBh-hSpCas9, 42230) and GFPmRNA was made using pCAG-GFP (Addgene, 11150). β-Catenin mRNA was created using pcDNA6-N-3XFLAG-Ctnnb1 plasmid (Addgene, 123586). Short DNA fragments including four point mutations that abolish β-catenin phosphorylation by GSK-3β S33A, S37A, T41A, S45A were ordered from IDT and cloned into pcDNA6-N-3XFLAG-Ctnnb1 plasmid using restriction enzyme digestion. Short DNA fragments, including four point mutations that abolish the association of β-catenin with Lef/Tcf factors K435A, R469A, H470A and K508A were ordered from IDT and cloned into pcDNA6-N-3XFLAG-Ctnnb1 plasmid using restriction enzyme digestion.

A DNA template for RNA synthesis was created using the forward and reverse primers listed in Table S3 with GXL prime star PrimeSTAR GXL DNA Polymerase (Takara, R050A). A DNA template for β-catenin mRNA synthesis was created using linearization of plasmid DNA downstream of the stop codon with restriction endonuclease XhoI (New England Biolab, R0146L). mMESSAGE mMACHINE T7 ULTRA Transcription Kit (Thermo Fisher Scientific Cat# AM1345) was used for *in vitro* mRNA synthesis from the DNA template to create 5′CAP 3′Polyadenylated tailed transcripts. Synthesized mRNA was precipitated by lithium chloride and run on a 1.5% agarose formaldehyde denaturing gel to validate proper size as well as tailing.

### Cell transfection

mRNAs as well as gRNAs were transfected into NPCs using Lipofectamine MessengerMAX Transfection Reagent (Thermo Fisher Scientific, LMRNA015). Per 24 wells, 500 ng of total mRNA (per transcript type) were added along with sgRNAs at 1 μM. Per 24 wells, 1.5 µg of β-catenin mRNA were added along with 500 ng of mCherry mRNA. NPCs were transfected according to manual instructions in OPTI-MEM. NPCs were incubated with OPTI-MEM for 3 h (see Fig. 2A).

### CRISPR mediated gene removal

gRNA targeting GFP were purchased from Thermo Fisher Scientific true guide gRNA sequence targeting exon 1 of GFP and designed using Invitrogen True Guide Tool. gRNAs targeting Ctnnb1 were designed using indephi to maximize frameshift potential (Shen et al., 2018) and cross referenced with Hodgkins et al. (2015) to minimize off target effects. We custom ordered Alt-R CRIPSR-Cas9 sgRNA, 2 nmol sgRNAs from IDT. We designed four guides, one targeting exon 2, two targeting exon 3 and one targeting exon 6 of β-catenin. See Table S3 for sgRNA sequences.

Lef/Tcf factor targeting using gRNAs used a 48 h KO period. gRNAs were ordered from Synthego and designed using CRISPR Design Tool based on the following criteria: (1) targeting an early coding exon, (2) targeting a common exon between all the transcripts, (3) high activity gene cutting and (4) minimal off target effects. The highest ranked top four guides were tested using immunofluorescence staining to measure protein loss as a metric of KO efficiency. Lyophilized guides were reconstituted at 100 pmol/µl in TE buffer and stored at −20°C. gRNAs were used at 7.5 pmol/well for a 24-well plate. We used 1.875 pmol/guide/well in case of quadruple KO (QKO) for Tcf7l1, Tcf7l2, Tcf7 and Lef1. sgRNAs were co-transfected into cells with mCherry (500 ng/well mRNA) using Lipofectamine MessengerMAX Transfection Reagent (Thermo Fisher Scientific, LMRNA015).

### RNA isolation and RT-qPCR

RNA was isolated with RNeasy micro kit (Qiagen, 74004). RNA was also isolated using this kit to send for bulk RNA-seq. For RT-qPCR, RNA was reverse transcribed in cDNA with SuperScript IV VILO Master Mix with ezDNase Enzyme (11766050). qPCR was performed using Luna Universal qPCR Master Mix Protocol (New England Biolab, M3003) on a ViiA 7 Real-Time PCR 96 System. See Table S3 for primer sequences.

### FACS sorting mRNA isolation

Before FACS sorting, NPCs were rinsed once with PBS, then incubated with trypsin for 5 min in the incubator at 37°C. The reaction was quenched with 10% FBS in AutoMACS buffer. NPCs were pelleted and washed once with AutoMACS buffer before resuspending AutoMACS with in DAPI [dead cell dye 4′,6-diamidino-2-phenylindole, dilactate (DAPI), 422801, BioLegend] and DRAQ5 (DRAQ5 Live cell dye, NBP2-81125-50 µl, Novus). 60,000 to 150,000 mCherry^+^ NPCs were sorted on a BD SORP FACS Aria Iiu into RLT buffer with 1:100 β-mercaptoethanol before RNA isolation.

### RNA-seq

Total RNA integrity was determined using Agilent Bioanalyzer or 4200 Tapestation. Library preparation was performed with 10 ng of total RNA with a Bioanalyzer RIN score greater than 8.0. ds-cDNA was prepared using the SMARTer Ultra Low RNA kit for Illumina Sequencing (Takara-Clontech) according to the manufacturer's protocol. cDNA was fragmented with a Covaris E220 sonicator using peak incident power 18, duty factor 20%, cycles per burst 50 for 120 s. cDNA was blunt ended, had an A base added to the 3′ ends, and then had Illumina sequencing adapters ligated to the ends. Ligated fragments were then amplified for 12-15 cycles using primers incorporating unique dual index tags. Fragments were sequenced on an Illumina NovaSeq-6000 using paired end reads extending 150 bases.

Basecalls and demultiplexing were performed with Illumina's bcl2fastq2 software. RNA-seq reads were then aligned to the combined mouse GRCm38 and human GRCh38 Ensembl release 76 primary assemblies with STAR version 2.5.1a1. Gene counts were derived from the number of uniquely aligned unambiguous reads by Subread:featureCount version 1.4.6-p52. Isoform expression of known Ensembl transcripts were estimated with Salmon version 0.8.23. Sequencing performance was assessed for the total number of aligned reads, total number of uniquely aligned reads and features detected. The ribosomal fraction, known junction saturation and read distribution over known gene models were quantified with RseQC version 2.6.24.

Normalized counts tables were run through DeSEQ2 (Love et al., 2014) to create differential gene expression tables with Log2FC cut-offs no less than 1 (no less than 0.5 Table S1.2) and *P*-adjusted values no greater than 0.05 can be found in Table S1. Significance of Cre and Cas9 intersected gene lists was calculated with hypergeometric function in R. Differential expression tables were passed through GO package clusterProfiler (Yu et al., 2012). Data were visualized using ggplot and complex heatmap functions in R (Gu, 2022). The Benjamini-Hochberg correction (False Discovery Rate) was applied as well as the significance associated with intersections compared with all genes expressed with gene normalized counts greater than or equal to 10 using hypergeometric test.

### Immunofluorescence staining

To perform immunostaining on NPCs in tissue culture, NPCs were cultured on coverslips (Thermanox plastic coverslip, Thermo Fisher Scientific, 174969), and cell cultures were fixed with 4% PFA in PBS for 10 min, then washed with PBS twice before blocking in 1.5% SEA block (Thermo Fisher Scientific, 107452659) in TBST (0.1% Tween-20 in TBS). After blocking at room temperature for 1 h, coverslips were switched to primary antibody (diluted in blocking reagent) incubation at 4°C overnight. After washing three times with TBST, we switched to secondary antibody (diluted in blocking reagent) incubation for 1 h at room temperature, shielded from light. This was followed by three washes with TBST and a final rinse in PBS. Cover slips were then flipped onto coverglass (VWR Micro Cover Glasses, Rectangular no 1.5 22×60, VWR 48393-221) onto 15 µl of mounting media [Fluoromount-G Mounting Medium (25 ml) Thermo Fisher Scientific, 00-4958-02]. After drying overnight, cover slips were taped with double-sided tape onto superfrost micro slides 25×75×1 mm (VWR, 48311-703). Slides were kept away from light at 4°C before confocal imaging. Primary and secondary antibodies used are listed in Table S3.

### EdU chasing

NPCs were chased with 10 μM EdU diluted in DMSO for 1 h before fixation with 4% PFA. To visualize EdU incorporation, we used Click-iT EdU Cell Proliferation Kit for Imaging, Alexa Fluor 647 dye (Thermo Fisher Scientific, C10340). NPCs were stained before the Click-it reaction.

### RNA-scope

To perform *in situ* fluorescent RNA analysis we used the RNA scope Multiplex Fluorescent Reagent Kit v2 (Advanced Cell Diagnostics). We followed the commercially available RNA-scope protocol: the tissue on slides was treated with hydrogen peroxide and protease, then hybridized with probes for 2 h at 40°C using a HybEZ oven (Advanced Cell Diagnostics). Probes were then amplified and detected with tyramide signal amplification fluorescence. The slides were incubated with 1 mg/ml Hoechst 33342 (Molecular Probes). The tissue was imaged with a 63× oil immersion lens using the Leica SP8 confocal microscope. The catalog numbers of probes from Advanced Cell Diagnostics can be found in Table S3.

### Image acquisition

Image acquisition was performed using Leica SP8-X confocal fluorescence imaging system (Leica Microsystems, Germany) in 1024×1024 pixels with a 63× or 40× Leica oil immersion objective (NA 1.6).

### Image quantifications

We quantified the confocal images of NPC experiments using Imaris microscopy image analysis software (version 9.9, Oxford Instruments).

#### β-Catenin KO quantification

We also quantified the membrane levels of β-catenin protein in the KO experiments using Imaris. Background noise was reduced applying the ‘Background Subtraction’ feature on the channel of examined protein. We manually created five membrane masks by the ‘surfaces’ module per image, measuring the fluorescent intensity of the shared membrane area between two adjacent transfected (KO) cells of the protein of interest, and calculated an average of these values. We chose to quantify membrane intensity only between two transfected cells to avoid the potential overestimation of protein levels: the signal of a non-transfected cell could overlap with the membrane of a KO cell. We also applied the same quantification method to adjacent non-transfected cells (the average of five membrane intensities between non-transfected cells). We calculated a ratio between the average fluorescent membrane intensity between two transfected cells and the average membrane intensity of non-transfected cells. This ratio has been multiplied by 100 and reported as the percentage KO of the protein. The latter normalization step was required to correct the differences in immunostaining and image acquisition across samples.

#### Jag1 loss from β-catenin KO quantification

Cells were manually classified as Jag1^+^ if the nuclear masks had granular Jag1 signal in their proximity and/or the signal was observed in membrane distribution.

#### EdU quantification

Cells were classified as mCherry^+^ or EdU^+^ with the use of spots module based on cut-offs of their respective fluorescent intensity histograms.

#### Six2 protein/Tcf7l1 protein level quantification in β-catenin KO

Six2 and Tcf7l1 intensity was determined based on the nuclear intensity values of cell module.

#### Tcf/Lef KO and loss of Jag1 protein with quadruple knockout quantification

The number of cells were automatically counted by the software using the DAPI nuclear marker after setting the appropriate nuclear radius. The cells were then classified as mCherry^+^ based on manual cut-offs of their respective fluorescent intensity histogram. These mCherry^+^ cells were further classified as Lef1^+^ based on manual cut-offs of their respective fluorescent intensity histogram. We calculated the decrease in protein levels of Lef1 by finding the ratio between the Lef1^+^ mCherry^+^ cells and total mCherry^+^ cells. This ratio has been multiplied by 100 and reported as the percentage KO of the protein. This quantification process was also applied to determine the decrease of protein levels of Tcf7, Tcf7l1, Tcf7l2 and Jag1.

Fluorescent intensity threshold was used for the ‘spots’ module for Lef1 and Six2 channels to classify Lef1^+^/Lef1^−^ and Six2^+^/Six2^−^ cells in β-catenin overexpression studies.

### Statistical analysis

Initially, the normal distribution of datasets was determined using a D'Agostino-Pearson test. In the case of normal distribution, the *P*-values were calculated using a Student's unpaired *t*-test. A Mann–Whitney test was applied for datasets with a non-normal distribution (*P*<0.05 was considered significant). In Figs 3 and 7, we used a D'Agostino-Pearson test as mentioned in figure legends and an ordinary one-way ANOVA (*P*<0.05 was considered significant). We quantified the individual and quadruple knockout (QKO) experiments using Imaris microscopy image analysis software (version 9.9, Oxford Instruments). Putative target genes of β-catenin and Tcf factors were predicted using GREAT (McLean et al., 2010) with the default setting, i.e. genes within 1 Mbp of the binding sites unless the nearest TSS was encountered (or more details, see http://great.stanford.edu/public/html/). The prediction produced Table S2. To test whether there is significant association between β-catenin/Tcf dependency and β-catenin binding, Fisher's exact test was carried out to evaluate the significance of enrichment of β-catenin association with 69 genes of the 90 genes dependent on Lef/Tcf interactions identified in QKO in high CHIR. GREAT was used for the total gene count (21,395) in mm10.

### Integration of scRNA-seq (mouse and human) and ChIP seq data with bulk RNA seq data

Previously published β-catenin and Tcf/Lef transcription factor ChIP-seq data (Guo et al., 2021) was downloaded from GEO (accession number GSE131117). Raw sequencing reads of input, Ctnnb1, Lef1, Tcf7 ChiP-seq from low/high-concentration (1.25 µM and 5 µM) CHIR-treated NPCs were aligned by Bowtie2 (47) on mm39 reference genome. Alignment files were sorted by samtools (Li et al., 2009) and filtered to remove duplicate reads using the picard ‘markduplicates’ tool (http://broadinstitute.github.io/picard/). Peak calling was performed by MACS2 with a q-value cut-off threshold of 10-8, to ensure strong transcription factor bindings in high-concentration CHIR treatment compared with low concentration counterparts. Peak annotation was performed by annotatePeaks.pl from the HOMER suite (Heinz et al., 2010) with default parameter. To incorporate any β-catenin/TCF/LEF downstream genes in the high-CHIR condition, the union set of annotated genes was used in the following intersection analysis. Downregulated DEGs in both Cas9- and Cre-mediated knockout bulk RNA-seq data were intersected with ChIP-seq peak associated genes. Finally, 161 genes in the intersection list were visualized with the Seurat (Stuart et al., 2019) FeaturePlot function in mouse post-neonatal day0 (P0) nephrogenic-lineage cells from kidney single-cell data (Kim et al., 2024). Total number of expressed genes in nephrogenic cells was calculated based on the threshold of more than 0.25% cells with normalized expression value of a gene.

### Human scRNA-seq analysis

To investigate conservation between mouse and human gene expression, the 161 genes in the intersection mouse ChIP list were visualized and homologs were intersected with Seurat FeaturePlot function in human fetal kidney (Lindström et al., 2021).

## Supplementary Material

10.1242/develop.202279_sup1 Supplementary information

Table S1. Data table including bulk mRNA Seq, sc-RNA-seq, ChIP analyses and intersections

Table S2. ChIP binding of indicated transcription (co)factors binding sites in E16.5 mouse nephron progenitor cells in the specified CHIR conditions

Table S3. Methods Table including primer sequences, antibodies and sgRNA sequences and RNA scope probes
