# Supplementary material for: Dose-dependent responses to canonical Wnt transcriptional complexes in the regulation of mammalian nephron progenitors
Source: Development. 2024 Sep 30;151(18):dev202279. doi: 10.1242/dev.202279 (PMC11463962; doi:10.1242/dev.202279)
Supplement: Supplementary information [file develop-151-202279-s1.pdf]

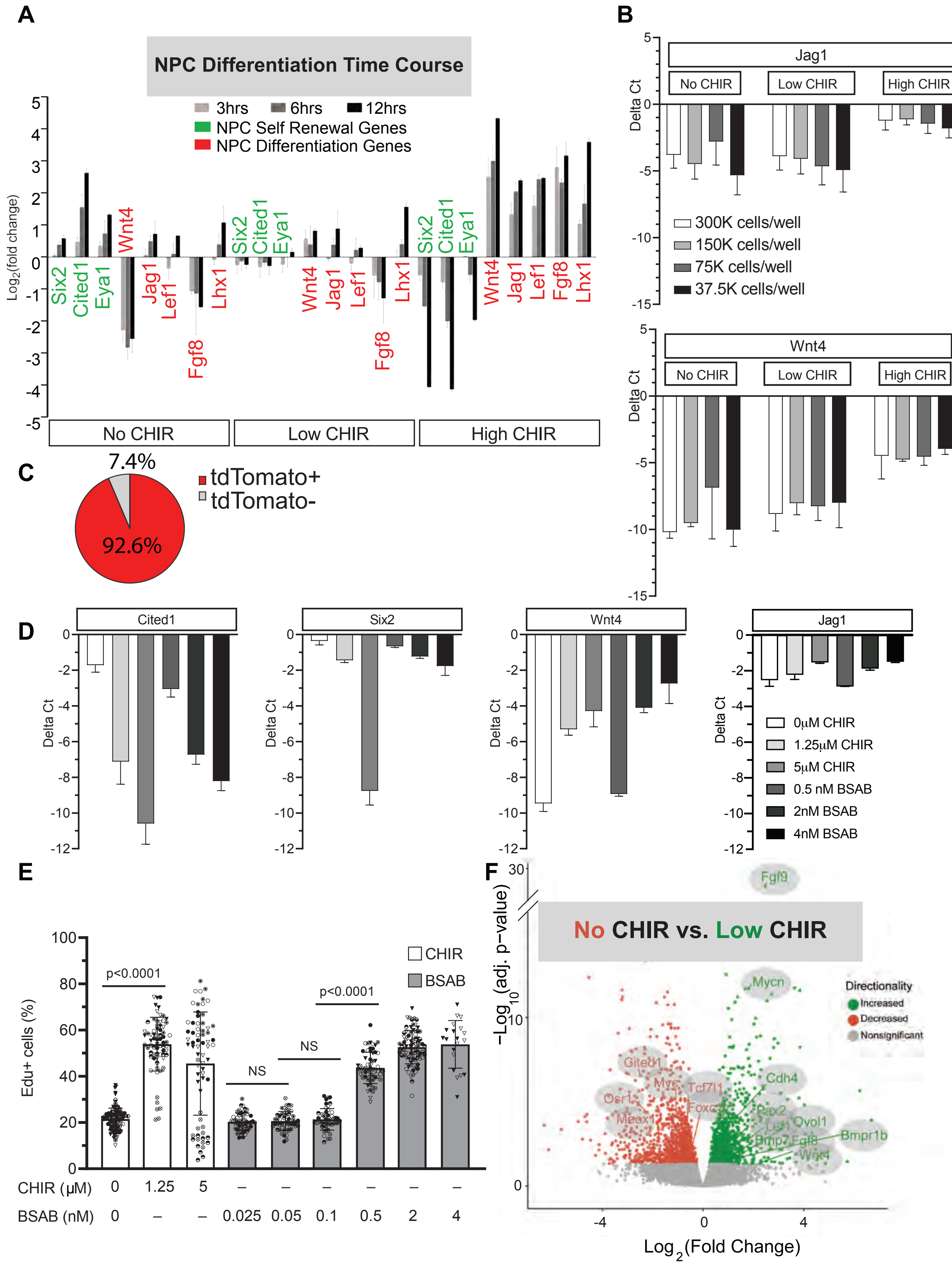

Development • Supplementary information

Fig. S1. Broad Characterization of NPC purity, gene induction timing, and proliferation with CHIR and surrogate Wnts.

A) RT-qPCR of NPC cultured across a 3, 6, 12h time course. Values are normalized to *Gapdh* and a ratio is calculated by dividing low CHIR condition values. Values are Log2 transformed. Green genes are self-renewing NPC markers and red genes are NPC differentiation markers. Grey panel denotes no CHIR, light gray denotes low CHIR and dark gray denotes high CHIR after a 24 h low CHIR seeding stabilization period (Biological replicates n= 3).

B) RT-qPCR results for Six2, Cited1, Wnt4, Lef1, Lhx1, Jag1 (Gapdh-Ct value) of NPC cultured at 300k, 150k, 75k and 37.5k at no, low and high CHIR. Values placed further from x-axis denote less expression. Delta Ct = (*Gapdh* – Ct gene value) (Biological replicates n= 3).

C) Percent purity of NPCs derived from Six2TGC-tdTomato mice. Purity measured as cell numbers of cells that are tdTomato + from Six2-Cre activity (Range from 86%-100% from 32 field of view from 8 wells.

D) RT-qPCR of self-renewal genes (Cited1, Six2) and induction genes (Wnt4, Jag1) of NPCs cultured with Wnt surrogate BSAB to validate the activation of Wnt pathway using CHIR. n= 2 biological replicates. ΔCt = (*Gapdh* – Ct gene value).

E) Quantification of EdU chasing (1h) of NPCs post 24-hour exposure to NPEM with different types and various levels of Wnt input (CHIR and BSAB, n=2-3 biological replicates), Mann-Whitney test, unpaired t-test.

F) Bulk RNA-seq data of DEGs using Log2FC = absolute value cut off = 0.5 and p-adjusted value cut off =0.05 comparing no CHIR and low CHIR post 24 hours of culture represented as a volcano plot.

Development • Supplementary information

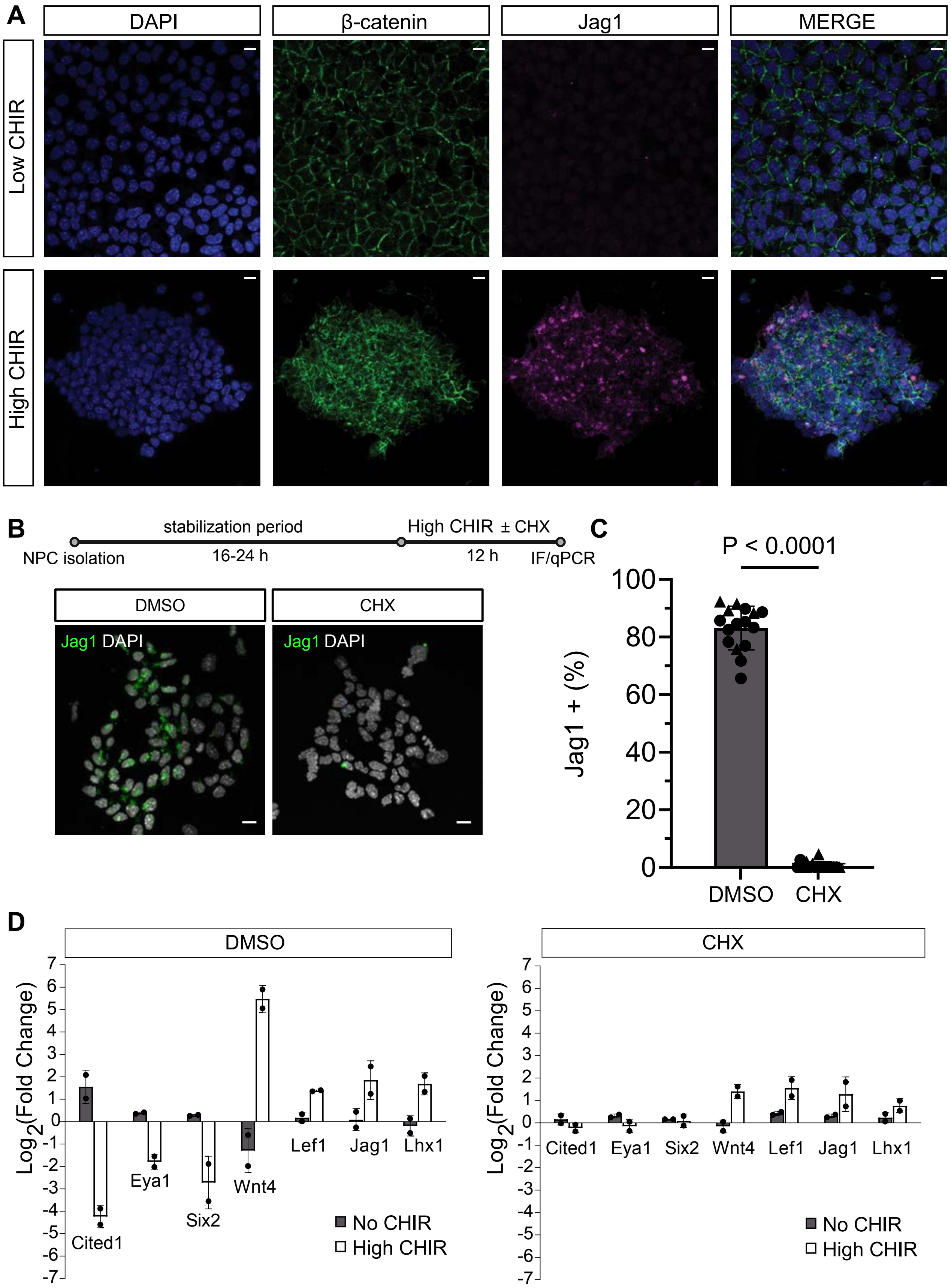

Fig. S2. Characterization of NPC protein expression and *de novo* protein synthesis

A) Immunofluorescent staining of β-catenin (green), Jag1 (purple), DAPI (blue) on wildtype NPC cultured in low and high CHIR (scale bar = 10 μm).

B) Immunofluorescent staining of NPCs treated with CHX for 12 h (scale bar = 10 μm).

F) Quantification of Jag1 expression in CHX treated NPCs vs. vehicle (DMSO) control. n=2 biological replicates denoted as different symbols, 16 field of views/group. Wilcoxon-test.

E) RT-qCPR of NPCs treated with CHX for 12h. Log2FC graphed compared to low CHIR conditions. Values normalized to *Gapdh* expression.

Development • Supplementary information

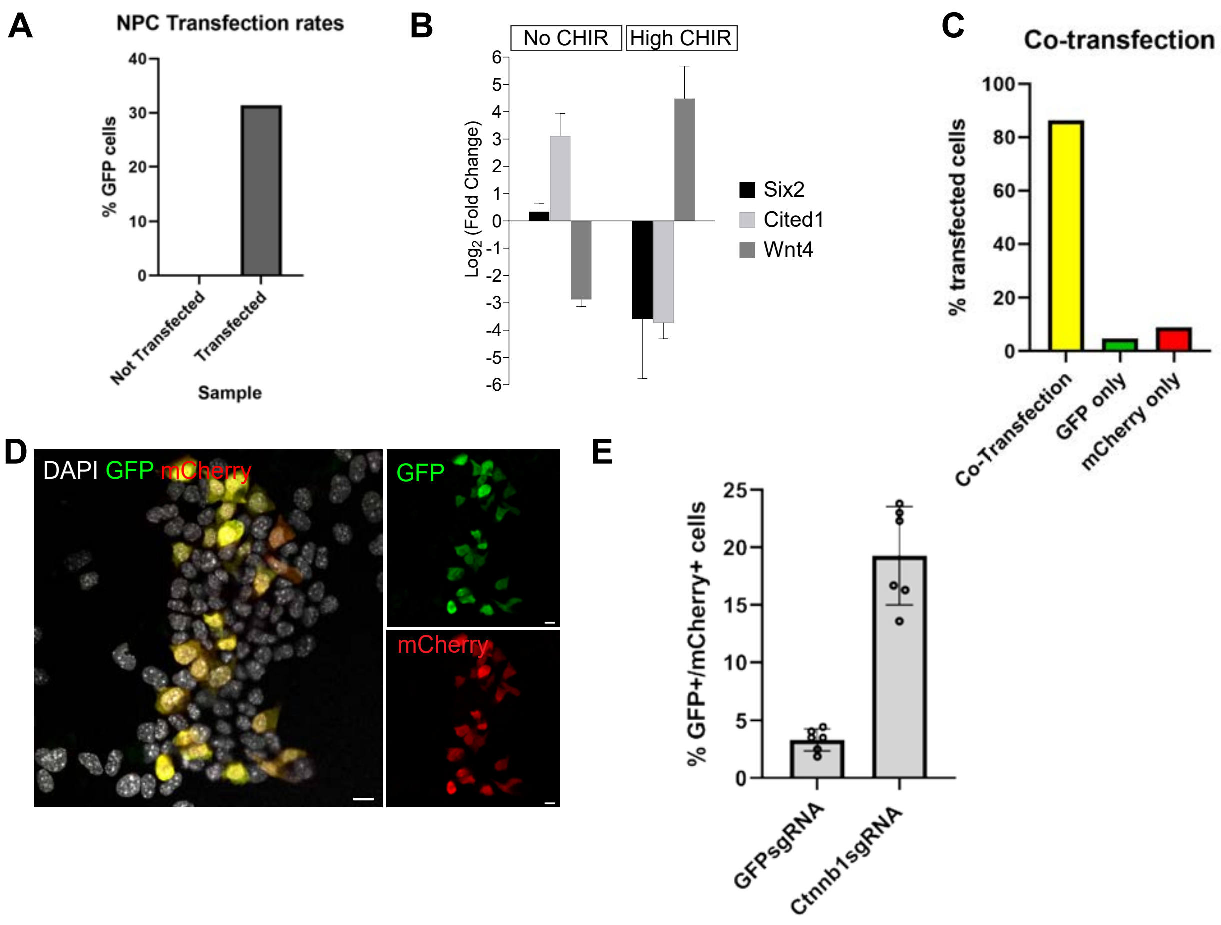

Fig. S3. Characterization of NPC mRNA transfection and gene editing methodology.

A) ransfection rates of GFP-mRNA in NPCs measured by FACS sorting (n=2 biological replicates)

B) -qPCR of NPCs transfected with GFP and harvested 24h after changing media with altering CHIR conditions. Ct-differences normalized to *Gapdh* and samples compared to low CHIR condition. Log2 transformed values are plotted. (n=2 biological replicates).

C) ACS sorting measuring co-transfection (mCherry mRNA and GFP mRNA) rates of mCherry and GFP mRNA into NPCs transfected together (co-transfection) or independently (n=2 biological replicates).

D) I munostaining of GFP and mCherry in NPCs demonstrating co-localization of GFP (green) and mCherry (red) appearing yellow (green + red) (scale bar = 10 µm).

E) FP positive NPCs in GFP sgRNA/mCherry co-transfected samples and Ctnnb1 sgRNA/mCherry co-transfected samples. GFP signal reduction from GFP sgRNA controls.

Development • Supplementary information

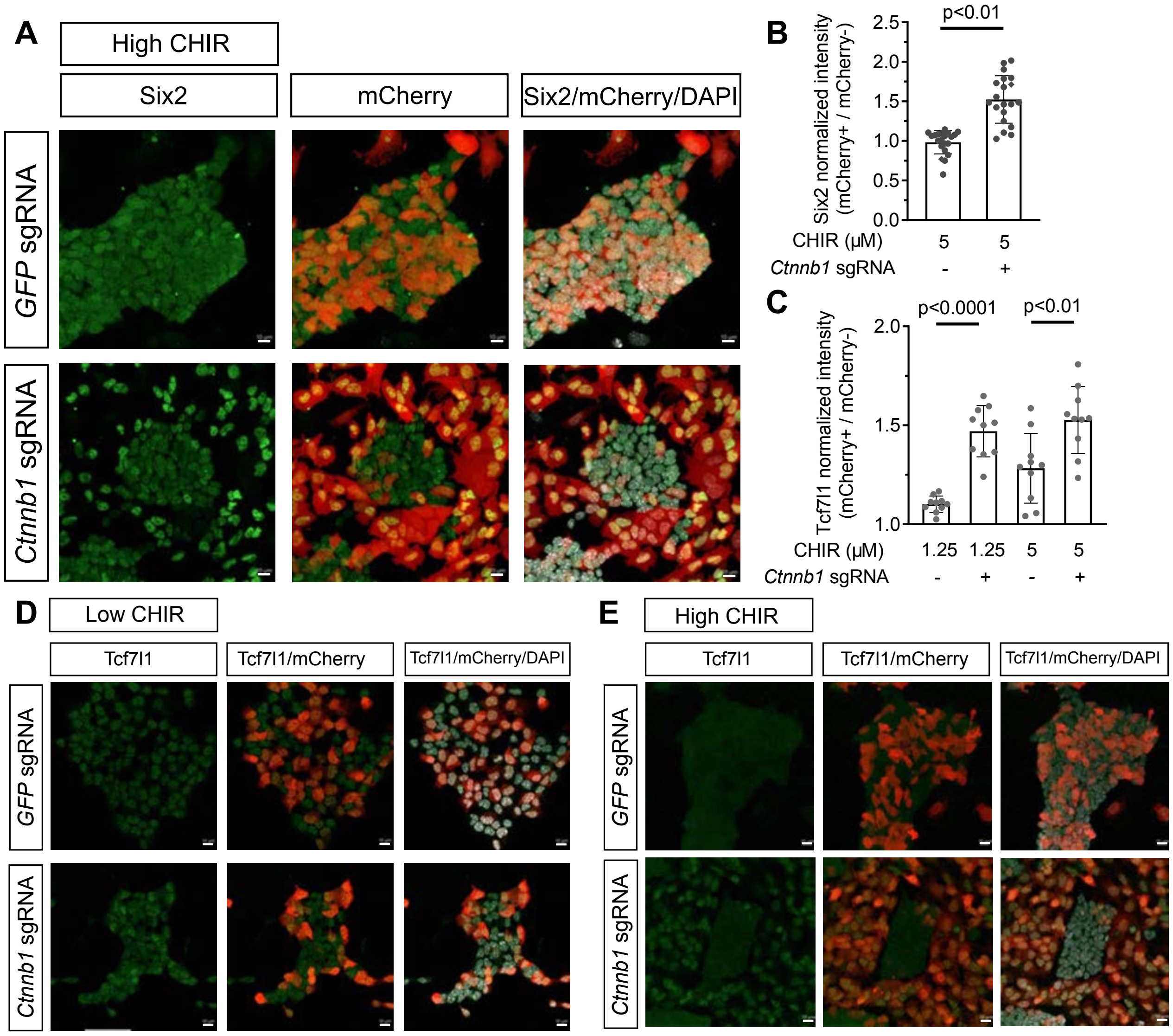

Fig. S4. Characterization of NPC Cas9 and Cre mediated protein removal in High CHIR

A) Immunostaining of NPCs with Cas9 mediated β-catenin KO in high CHIR.

B) Quantification of the increase in Six2 immunofluorescent intensity in Cas9 mediated β-catenin removal of NPCs cultured in high CHIR. Unpaired t test. 4-8 fields of view,3 biol repl, normality test pass, Student t-test.

C) Quantification of increase in Tcf7l1 staining in Cre mediated β-catenin removal of NPCs Unpaired t test. 10 fields of view,1 technical replicate, normality test pass, Student t-test.

D) Immunostaining of NPCs cultured in 1.25 uM CHIR. Tcf7l1= green, mCherry = Red.

E) Immunostaining of NPCs cultured in 5uM CHIR. Tcf7l1= green, mCherry = Red

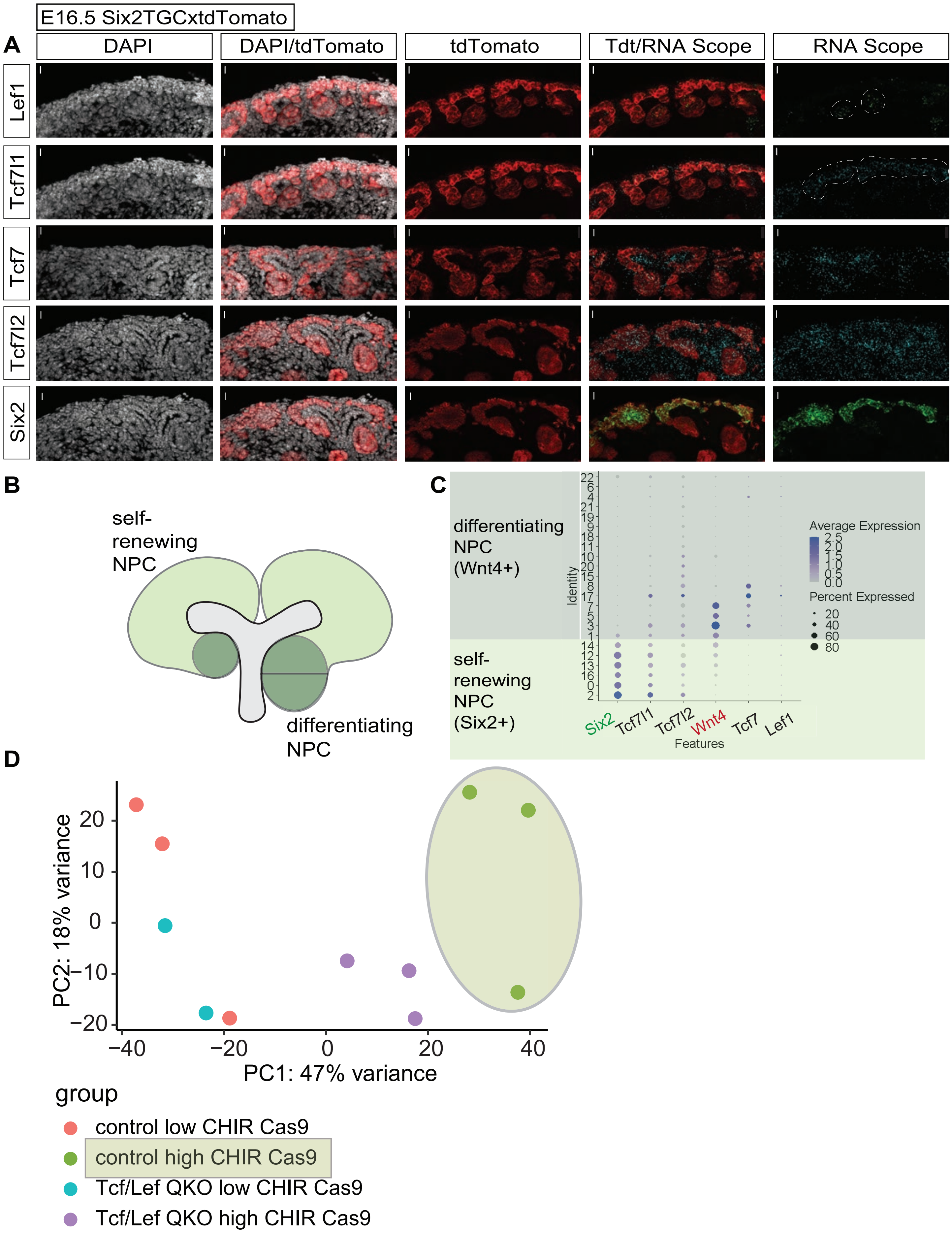
E16.5 Six2TGCxtdTomato
A
DAPI
DAPI/tdTomato
tdTomato
Tdt/RNA Scope
RNA Scope
Lef1
Tcf7l1
Tcf7
Tcf7l2
Six2
B
self-renewing NPC
differentiating NPC
C
differentiating NPC (Wnt4+)
self-renewing NPC (Six2+)
Identity
Average Expression
Percent Expressed
20
40
60
80
Six2
Tcf7l1
Tcf7l2
Wnt4
Tcf7
Lef1
Features
D
PC2: 18% variance
PC1: 47% variance
20
10
0
−10
−20
−40
−20
0
20
40
group
control low CHIR Cas9
control high CHIR Cas9
Tcf/Lef QKO low CHIR Cas9
Tcf/Lef QKO high CHIR Cas9

Fig. S5. ***In Vivo*** **characterization of** ***Tcf/Lef*** **factor gene expression in self-renewing and differentiating niches in Kidney Development**

A) RNA scope of E16.5 Six2TGC x Tdt kidneys. Cyan = Tcf7l1, Lef1= Green, tdT antibody = red, DAPI= white.

B) RNA scope of E16.5 Six2TGC x Tdt kidneys. Cyan = Tcf7l2, DAPI= white, tdT antibody = red

C) RNA scope of E16.5 Six2TGC x Tdt kidneys. Cyan = Tcf7, Lef1 antibody = Green, tdT antibody = red, DAPI= white.

D) scRNA-seq E16.5 kidneys – (16) dotplot showing Tcf7l1 and Lef1 expression in self-renewing and induced NPCs respectively. Anchor genes Six (green) denotes self-renewing NPCs and Wnt4 (red) denotes induced NPCs.

E) PCA plot of control (GFP sgRNA) and QKO (Tcf7l1, Tcf7l2, Tcf7, Lef1 sgRNA) in low and high CHIR

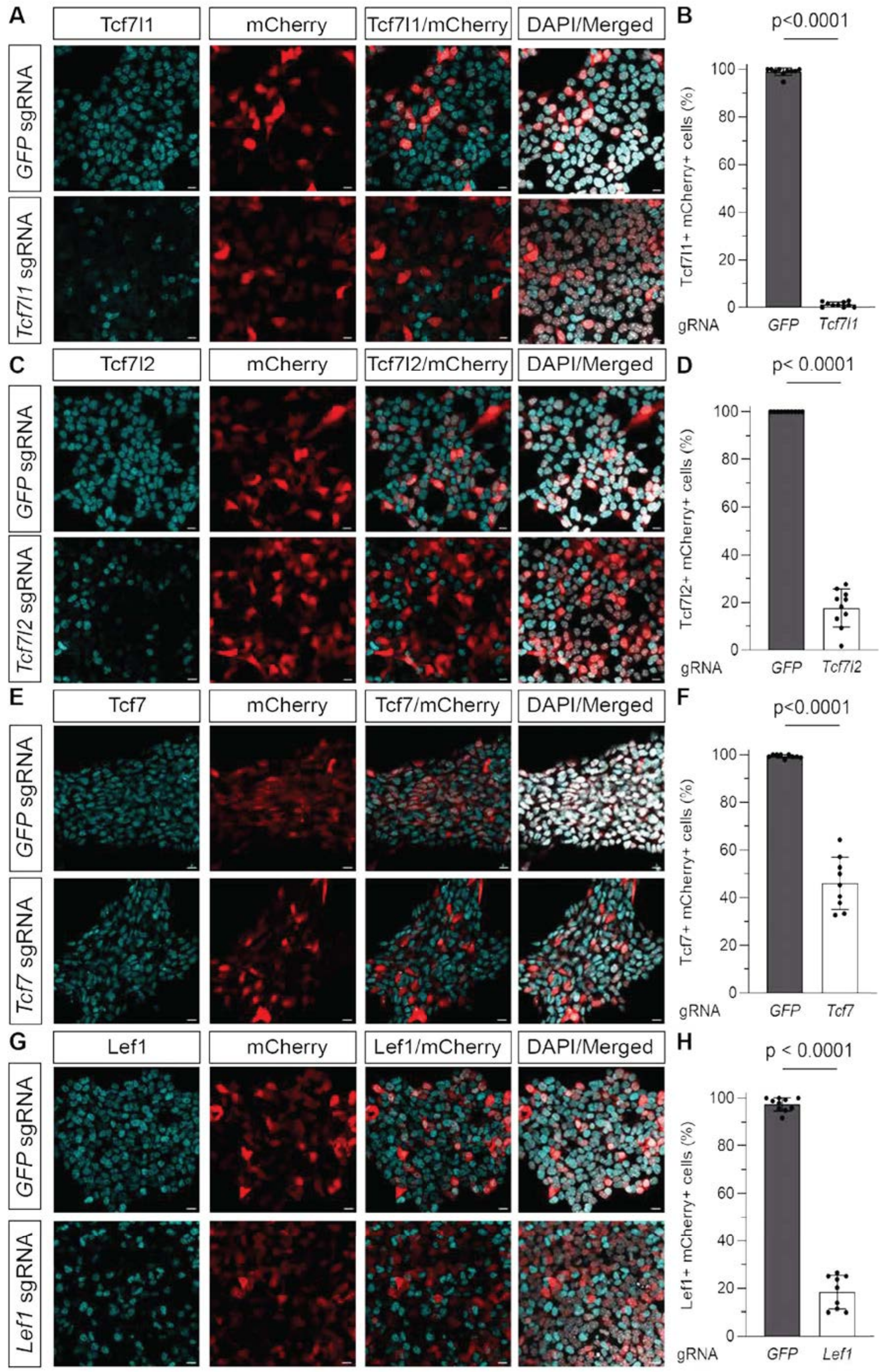

Development • Supplementary information

Fig. S6. Characterization of Cas9 mediated Tcf/Lef factor removal in NPCs

A) Immunofluorescence staining denoting Tcf7l1 protein removal (10µm scale bar).

B) Quantification of Tcf7l1 protein removal. Mann-Whitney test, 10 field of views/well.

C) Immunofluorescence staining denoting Tcf7l2 protein removal (10µm scale bar).

D) Quantification of Tcf7l2 protein removal. 10 field of views/well. Unpaired t test.

E) Immunofluorescence staining denoting Tcf7 protein removal (10µm scale bar).

F) Quantification of Tcf7 protein removal., 9 field of views/well. Unpaired t test.

G) Immunofluorescence staining denoting Lef1 protein removal (10µm scale bar).

H) Quantification of Lef1 protein removal. 9-10 field of views/well. Unpaired t test.

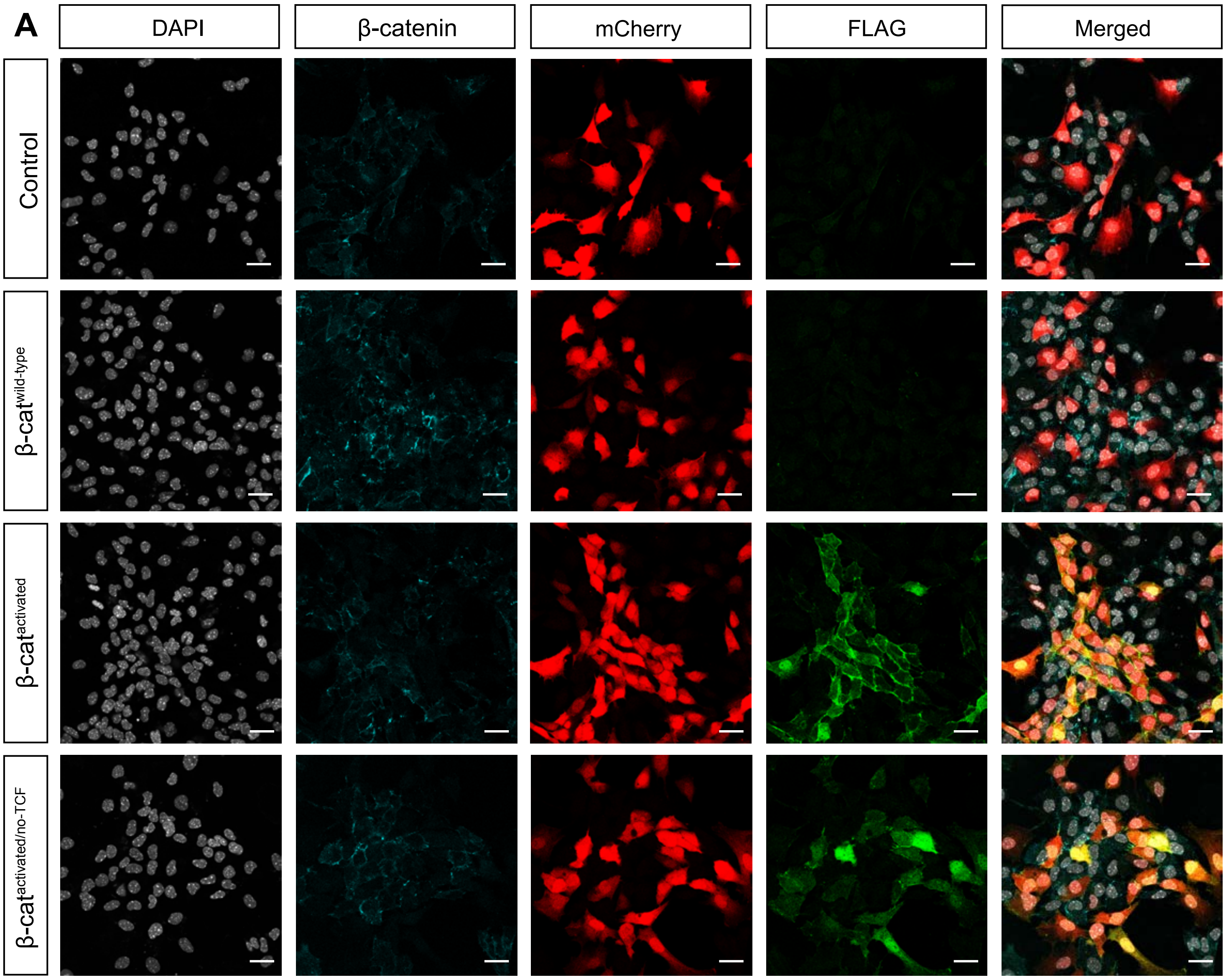

Fig. S7. FLAG-immunostaining of wild-type β-catenin and two activated forms (βCat^{activated} & βCat$^{activated/no-Tcf}$) of β-catenin in transfected cells

A) Immunostaining of NPCs cultured in low CHIR. DAPI= White, β-catenin = Cyan, mCherry = Red, FLAG = Green. 25uM Scale Bar.

Development • Supplementary information

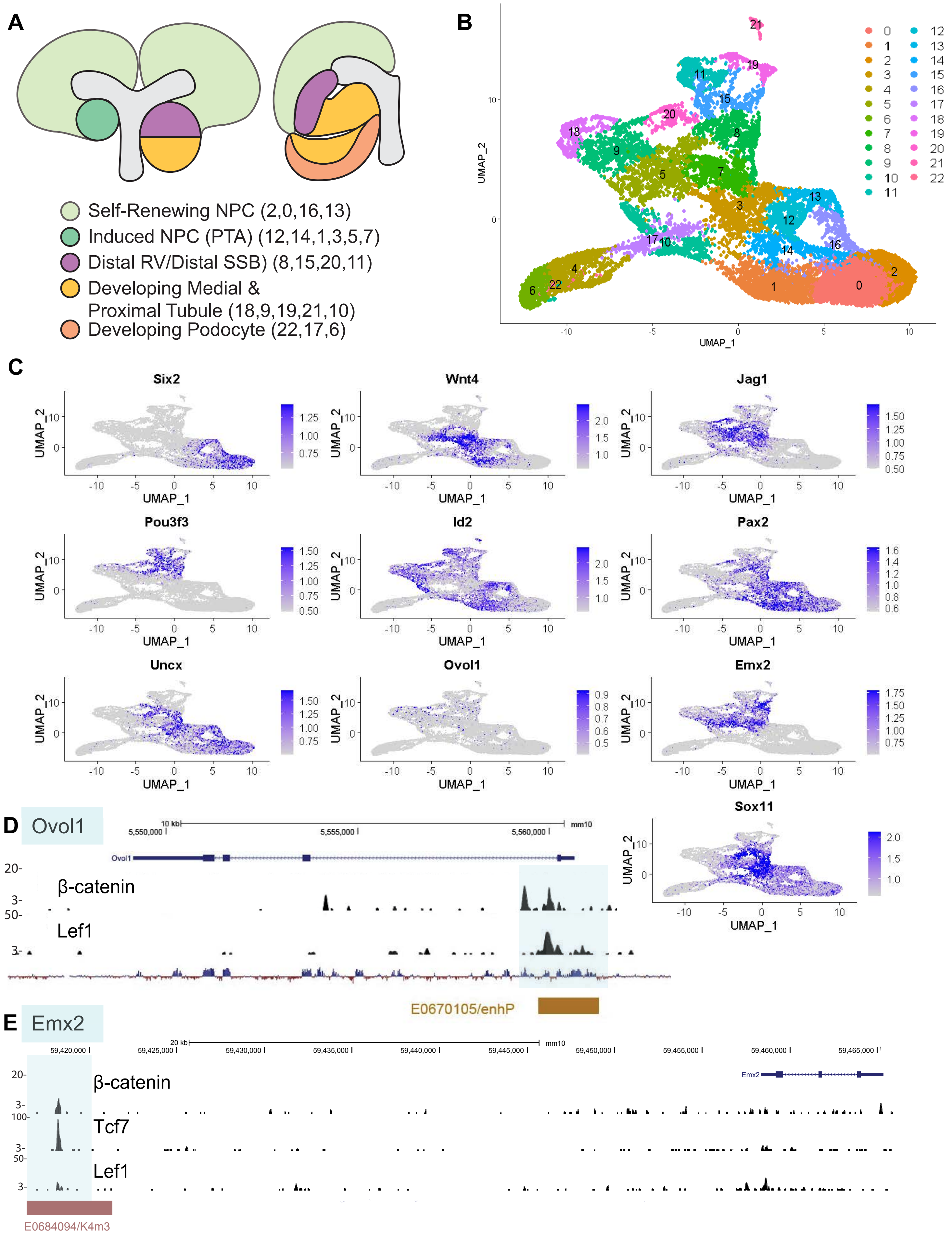
A
Self-Renewing NPC (2,0,16,13)
Induced NPC (PTA) (12,14,1,3,5,7)
Distal RV/Distal SSB) (8,15,20,11)
Developing Medial &
Proximal Tubule (18,9,19,21,10)
Developing Podocyte (22,17,6)
B
UMAP_1
UMAP_2
C
Six2
Wnt4
Jag1
Pou3f3
Id2
Pax2
Uncx
Ovol1
Emx2
Sox11
D
Ovol1
β-catenin
Lef1
E0670105/enhP
E
Emx2
β-catenin
Tcf7
Lef1
E0684094/K4m3

Fig. S8. Characterization of key differentiation markers *in vivo* and ChIP seq binding of Tcf/Lef/β-catenin at key target genes

A) Schematic representation of mouse nephrogenic niche and a developing s-shape body

B) MAP of all clusters of nephrogenic linages of cells from p0 scRNA-seq data (Kim et al., 2024)

C) Feature plots of β-catenin target genes *Pax2, Id2, Uncx, Emx2, Ovol1, Dach1, Sox11* with known markers of self-renewing NPCs (Six2, Cited1), early induction gene (Wnt4), Distal marker (Pou3f3), proximal tubule marker (Hnf4a), podocyte gene (Mafb) in mouse p0 scRNA-seq

D) Gene tracks of ChIP-seq data at Ovol1 locus showing β-catenin/ Lef1 binding at annotated cis-regulatory module of *Ovol*1 gene

E) Gene tracks of ChIP-seq data at *Emx2* locus showing β-catenin/ Lef1/Tcf7 binding at annotated cis-regulatory module of *Emx2* gene

Table S1. Data table including bulk mRNA Seq, sc-RNA-seq, ChIP analyses and intersections

Available for download at
https://journals.biologists.com/dev/article-lookup/doi/10.1242/dev.202279#supplementary-data

Table S2. ChIP binding of indicated transcription (co)factors binding sites in E16.5 mouse nephron progenitor cells in the specified CHIR conditions

Available for download at
https://journals.biologists.com/dev/article-lookup/doi/10.1242/dev.202279#supplementary-data

Table S3. Methods Table including primer sequences, antibodies and sgRNA sequences and RNA scope probes

Available for download at
https://journals.biologists.com/dev/article-lookup/doi/10.1242/dev.202279#supplementary-data
